# Design and selection of peptides to block the SARS-CoV-2 receptor binding domain by molecular docking

**DOI:** 10.3762/bjnano.13.62

**Published:** 2022-07-22

**Authors:** Kendra Ramirez-Acosta, Ivan A Rosales-Fuerte, J Eduardo Perez-Sanchez, Alfredo Nuñez-Rivera, Josue Juarez, Ruben D Cadena-Nava

**Affiliations:** 1 Centro de Nanociencias y Nanotecnología - Universidad Nacional Autónoma de México (UNAM) – Ensenada, Baja California, México; 2 Centro de Investigación Científica y de Educación Superior de Ensenada, Baja California, (CICESE), Ensenada, Baja California, México; 3 Departamento de Física, Universidad de Sonora, Blvd. Luis Encinas y Rosales, Hermosillo, Sonora, México

**Keywords:** angiotensin converting enzyme-2 (ACE2), antiviral peptides, hydrogen bonds, molecular docking, SARS-CoV-2 RBD

## Abstract

The novel Severe Acute Respiratory Syndrome Coronavirus-2 (SARS-CoV-2) is currently one of the most contagious viruses in existence and the cause of the worst pandemic in this century, COVID-19. SARS-CoV-2 infection begins with the recognition of the cellular receptor angiotensin converting enzyme-2 by its spike glycoprotein receptor-binding domain (RBD). Thus, the use of small peptides to neutralize the infective mechanism of SARS-CoV-2 through the RBD is an interesting strategy. The binding ability of 104 peptides (University of Nebraska Medical Center’s Antimicrobial Peptide Database) to the RBD was assessed using molecular docking. Based on the molecular docking results, peptides with great affinity to the RBD were selected. The most common amino acids involved in the recognition of the RBD were identified to design novel peptides based on the number of hydrogen bonds that were formed. At physiological pH, these peptides are almost neutral and soluble in aqueous media. Interestingly, several peptides showed the capability to bind to the active surface area of the RBD of the Wuhan strain, as well as to the RBD of the Delta variant and other SARS-Cov-2 variants. Therefore, these peptides have promising potential in the treatment of the COVID-19 disease caused by different variants of SARS-CoV-2. This research work will be focused on the molecular docking of peptides by molecular dynamics, in addition to an analysis of the possible interaction of these peptides with physiological proteins. This methodology could be extended to design peptides that are active against other viruses.

## Introduction

**Figure 1:**
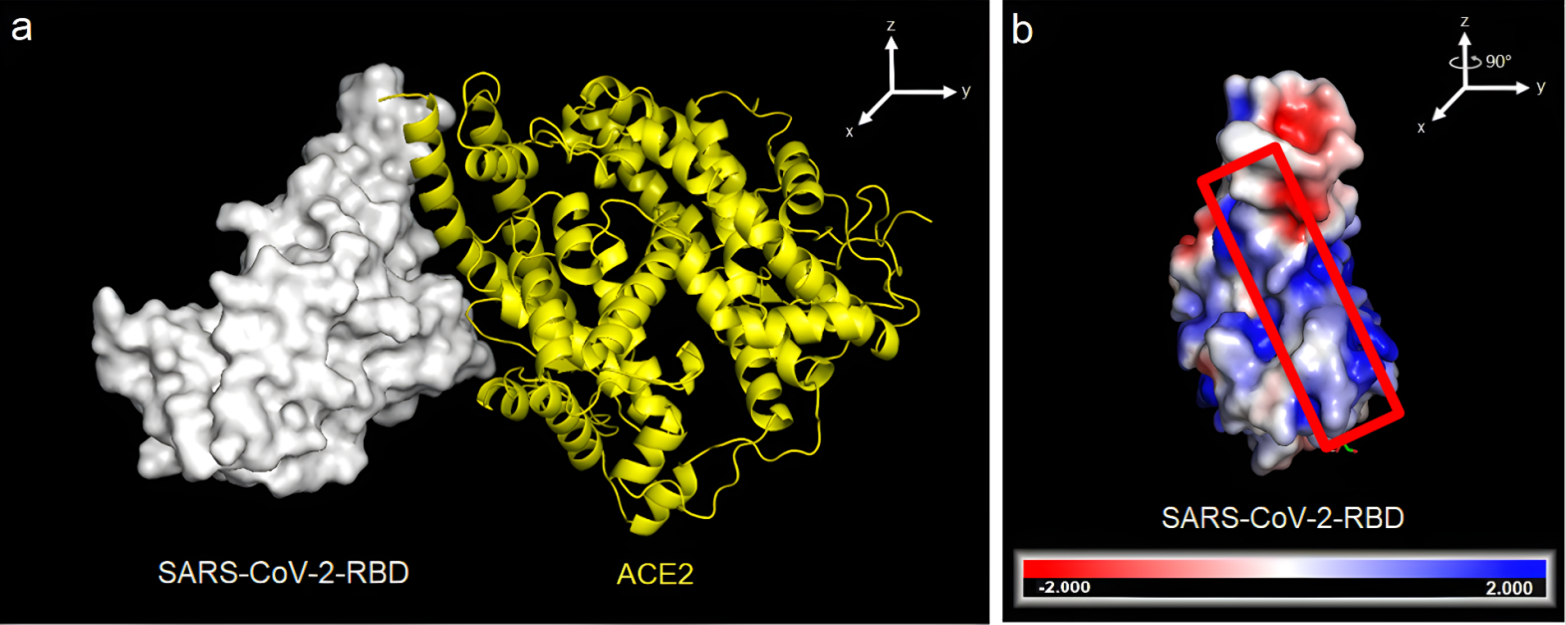
SARS-CoV-2 bound to the ACE2 receptor. (a) Crystallized complex between the RBD of the SARS-CoV-2 spike protein (white) and ACE2 (yellow) (PDB 6VYB). (b) Distribution of electrostatic potential on the surface of the SARS-CoV-2 spike protein. The electrostatic potential distribution was calculated using the adaptive Poisson–Boltzmann solver (APBS) module in PyMOL. The values range from −2 (red) over 0 (white) to +2 (blue). The orientation of the molecule is rotated by about 90° along the *z*-axis of image (a) to show the RBD surface that binds to the ACE2 cell receptor. Image modified from PDB 6VYB [3].

The current pandemic due to coronavirus disease-19 (COVID-19), caused by the novel virus SARS-CoV-2, has over 533 million of confirmed cases and over 6.3 million fatalities over the five continents by June 15, 2022 [1]. It is known, that the entry of the SARS-CoV into the host cell begins with the binding of the RBD, which is part of the spike (S) glycoprotein, to the angiotensin converting enzyme-2 (ACE2) cellular receptor of the host cells [2–4]. Similarly, the entry of SARS-CoV-2 into cells is mediated by the interaction of the RBD with the host cell ACE2 receptor [2–3,5]. Figure 1 shows the RBD–ACE2 complex formed at the first stage of the cellular infection by SARS-CoV-2. The formation of the RBD–ACE2 complex is mediated by the amino acid residues F486, Y489, Q493, G496, T500, and N501 located on the active region of the SARS-CoV-2 RBD (Figure 1b, red rectangle) [6]. Therefore, the RBD has been proposed as one of therapeutic targets to block the infection mechanism of SARS-CoV-2. For instance, peptide analogues of the ACE2 receptor (I21 to S44) have been designed in silico to disrupt the formation of the RBD–ACE2 complex and to prevent the viral infection [7].

Small peptides (biological and synthetic) have been proposed as promising alternative drugs to block the SARS-CoV-2 RBD and to interrupt the infection [8]. Lactoferricin B, minidefensins, indolicidin, and dermaseptin peptides have been used to neutralize viruses such as human immunodeficiency virus, cytomegalovirus, herpes simplex virus, and hepatitis B virus [9–11]. In order to find an effective peptide, it is important to reduce secondary effects by avoiding the binding with the major histocompatibility complex (MHC). This is crucial to reduce any acute immunological responses [12–13]. Several suitable peptide candidates could be found to block the SARS-CoV-2 RBD. Natural antiviral and antimicrobial peptides and chimeric peptides with the capability to bind and neutralize viral proteins can be designed and selected by phage display or using in silico approaches [8,14]. Several peptides based on the ACE2 receptor have been designed by in silico approaches [5,15]. In silico approaches are commonly used to determine the capacity of small ligands (peptides and drugs) to bind to a particular target site of a given protein, due to the low cost, versatility, and ease to develop [16–18]. Current peptide design techniques involve the modification of peptides based on the ACE2 receptor to increase their binding affinity to the SARS-CoV-2 RBD and to prevent the virus from binding onto the ACE2 receptor [5,15].

Powerful computational programs, as local installations or on internet servers, can be used to perform molecular docking. These include DOCK, AutoDock, FlexX, SurFlex, GOLD, ICM, and AutoDock Vina [18–20]. AutoDock Vina (ADV), an open-source software with high docking power, is one of the most commonly used programs [16–17,21]. ADV provides theoretical information about hydrophobic interactions, electrostatic interactions, hydrogen bonds, and van der Waals interactions. It can also predict the binding pose and binding affinity [20]. Considering these important features, ADV was used to perform molecular docking of 104 biological peptides, selected from the University of Nebraska Medical Center’s Antimicrobial Peptide Database (APD) [22–24], and theoretical peptides with the region of the SARS-CoV-2 RBD that binds to the cellular receptor ACE2 (called the RBD active region). APD peptides were selected based on an already known antiviral activity (Table S1, Supporting Information File 1). ADV results showed that both peptides from the APD and theoretical peptides have the capability to dock on the RBD active region, blocking the amino acid residues related to the association of the Wuhan strain RBD (GenBank: MN908947.3) with ACE2 [6]. The number of hydrogen bonds between RBD and ACE2 influence the stability of the bound complex, which suggests that designing peptides capable of forming several hydrogen bonds might prove useful for increasing the binding affinity [25]. From this, new theoretical peptides were designed, considering the most common amino acid residues involved in the formation of hydrogen bonds to the RBD active region of SARS-CoV-2. These small peptides may reinforce and enhance the effectiveness of the immune system response before and after the application of SARS-CoV-2 vaccines [26–27]. Furthermore, the proposed peptides may elude the immune system and bind effectively to SARS-CoV-2 RBD.

## Materials and Methods

### Ligand selection for screening

In total, 104 peptides were chosen from the University of Nebraska Medical Center’s Antimicrobial Peptide Database [22–24]. The peptides were selected based on their previously reported antiviral activity in order to perform a massive docking experiment. PDB files were gathered for those peptides that possess a reported three-dimensional structure and a 3D structure was predicted through I-TASSER’s and PEP-FOLD3.5’s model prediction servers using the reported peptide sequence for those that lacked it [28–32]. All peptides analyzed were compared with the ACE2-derived peptide IEEQAKTFLDKFNHEAEDLFYQSS (I21 to S44 of ACE2) [7]. The electrostatic surface potential, hydrophobic interactions, hydrogen bonds, and interactions of the selected ligand and protein docked complexes were analyzed by PyMOL (version 2.4) and LigPlot+ (version 2.2) [33–34].

### Protein and ligand preparation for AutoDock Vina

AutoDock Vina (1.1.2) software was employed for all docking experiments [21]. The X-ray diffraction crystal structure of SARS-CoV-2 RBD spike protein (PDB ID: 6VYB) was selected for this study. The molecules bound to the protein receptor molecule were removed. The RBD spike protein was prepared using AutoDock Tools to add polar hydrogen atoms, Kollman charges, and to remove water molecules. The active site grid was generated using a grid box (22 Å × 40 Å × 40 Å) centered at (11 Å, 90.5 Å, 57.5 Å). Docking was carried out with the 104 peptides from the APD as ligand molecules. The experiment was validated by comparing the position of the docked ACE2–RBD complex with the crystallized ACE2–RBD complex. The docked complex was superimposed onto the crystallized complex using PyMOL and an RMSD calculation was performed.

### Proposing new peptides based on hydrogen-bond formation

Based on the selected APD peptides, new peptides were designed in order to improve the capability to block the SARS-CoV-2 RBD. The standard way to design novel peptides is by random sequences that can generate many peptides. However, this leads to an increase in computation time. To avoid this bottleneck, peptides were designed through the analysis of the APD–RBD active region docking, where the RBD amino acid residues able to form hydrogen bonds with the APD were considered. Five main regions (vertical, horizontal, left diagonal, right diagonal, center) in the RBD active region were selected to propose the sequence of amino acid residues of a theoretical peptide that could potentially form a great number of hydrogen bonds in these positions while docked to SARS-CoV-2 RBD. The proposed sequences of amino acid residues were modeled using PEP-FOLD 3.5 server. Then, theoretical peptides were submitted to molecular docking against the Wuhan strain (PDB ID: 6VYB), Delta variant (PDB ID: 7W92) and theoretical variants (K417N, Y453F, E484K, and N501Y), according to the B1.1.7, B.1.351, P.1, and Y453F SARS-CoV-2 variants.

### Computing the radius of gyration

The radius of gyration (*R*_g_) for the selected peptides against SARS-CoV-2 was determined using the WinHydroPro V10 software [35].

### Immunogenicity analysis

Immunogenicity analysis of the selected peptides was carried out by the Tepitool software using specific alleles from the human major histocompatibility complex class I (MHC I) [36]. Peptides with low binding to MHC I molecules were considered (50 nM ≤ IC_50_ ≤ 500 nM) according to Calis and Adhikari [37–38]

### Free energy of RBD–ligand by PRODIGY

The protein binding energy prediction (PRODIGY) web server is an effective predictive model based on intermolecular contacts in protein–protein complexes based on their 3D structure [39]. This tool predicts the binding free energy between protein complexes with great accuracy, which makes it an excellent complement to docking approaches. Thus, PRODIGY web server was used to predict the binding energy of the APD–RBD and theoretical peptide–RBD complexes [39–41]. The input files were acquired from ADT files in the PDB format.

### Contact area analysis

PyMOL was used to compute the solvent-accessible surface area of SARS-CoV-2 RBD and the peptides that were docked to it. The surface area of the RBD–ligand complex was also calculated. The following equation was established to calculate the contact area between the RBD and each ligand analyzed:


[1] $$A_{\text{c}}=\frac{A_{\text{RBD}}+A_{\text{ligand}}-A_{\text{complex}}}{2},$$

where *A*_c_ is the contact area of the ligand with the RBD, and *A*_RBD_ and *A*_ligand_ indicate the surface areas of RBD and ligand, respectively. *A*_complex_ corresponds to the surface area of the complex formed when the ligand binds to the SARS-CoV-2 RBD.

## Results and Discussion

### Initial virtual screening

Based on the binding affinity obtained through ADV, 69 of the 104 APD peptides bound stronger to the RBD active region than the ACE2 peptide (Table 1 and Table S2, Supporting Information File 1). Three peptides based on lysozyme were also designed for screening to compare with the APD peptides given the antimicrobial role of lysozyme as part of the innate immune system. The ADV results show that most of the APD peptides successfully docked on the active region of the RBD (Figure 1), suggesting that these APD peptides actually bind to the RBD active region, blocking the entry of SARS-CoV-2 to host cells. Additionally, according to Figure 1, peptides are posed in different ways on the RBD, covering different areas of the active surface of RBD. For instance, MVL (74–87), cyanovirin-N (70–80) and dermaseptin-S4 (Figure S2, Supporting Information File 1) are posed laterally to the RBD active surface. Similar results have been reported previously by Qiao & Olvera, who designed a negatively charged EELE tetrapeptide to neutralize the SARS-CoV-2-RBD–ACE2 binding [25]. It is important to note that the analyzed peptides have a nearly neutral charge, thus they have a low probability of unspecific interactions with other molecules, cellular uptake, or macrophage recognition [42–46].

**Table 1:** Potential peptide candidates against SARS-CoV-2 obtained by molecular docking. The table shows the physical and biochemical properties of the potential peptides.

Peptide	PDB/UNIPROT ID	Residues	Number of amino acids	H bonds/residue	Affinity (kcal/mol)

alpha basrubrin	P83186	1–20	20	0.95	−5.2
human beta defensin 3	1KJ6	27–44	19	0.95	−5.0
sesquin	P84868	1–10	10	0.80	−5.6
indolicidin	1G89	1–13	13	0.77	−8.0
GF-17	2L5M	1–17	17	0.76	−5.3
cyanovirin-N (70–80)	2EZM	70–80	10	0.73	−5.3
protegrin 5	2NC7	1–18	18	0.72	−7.2
MVL (94–110)	1ZHS	94–110	17	0.71	−5.0
temporin B	6GIL	1–13	13	0.69	−5.6
dermaseptin-S4	2DD6	1–13	13	0.69	−5.5
MVL (74–87)	1ZHS	74–87	14	0.64	−5.9
MVL (16–34)	1ZHS	16–34	19	0.63	−5.8
ACE2	6VYB	21–44	24	0.63	−4.6
lysozyme (1–20)	1REX	20	20	0.35	−4.9
lysozyme (61–80)	1REX	20	20	0.60	−5.7
lysozyme (111–130)	1REX	20	20	0.25	−5.2

**Figure 2:**
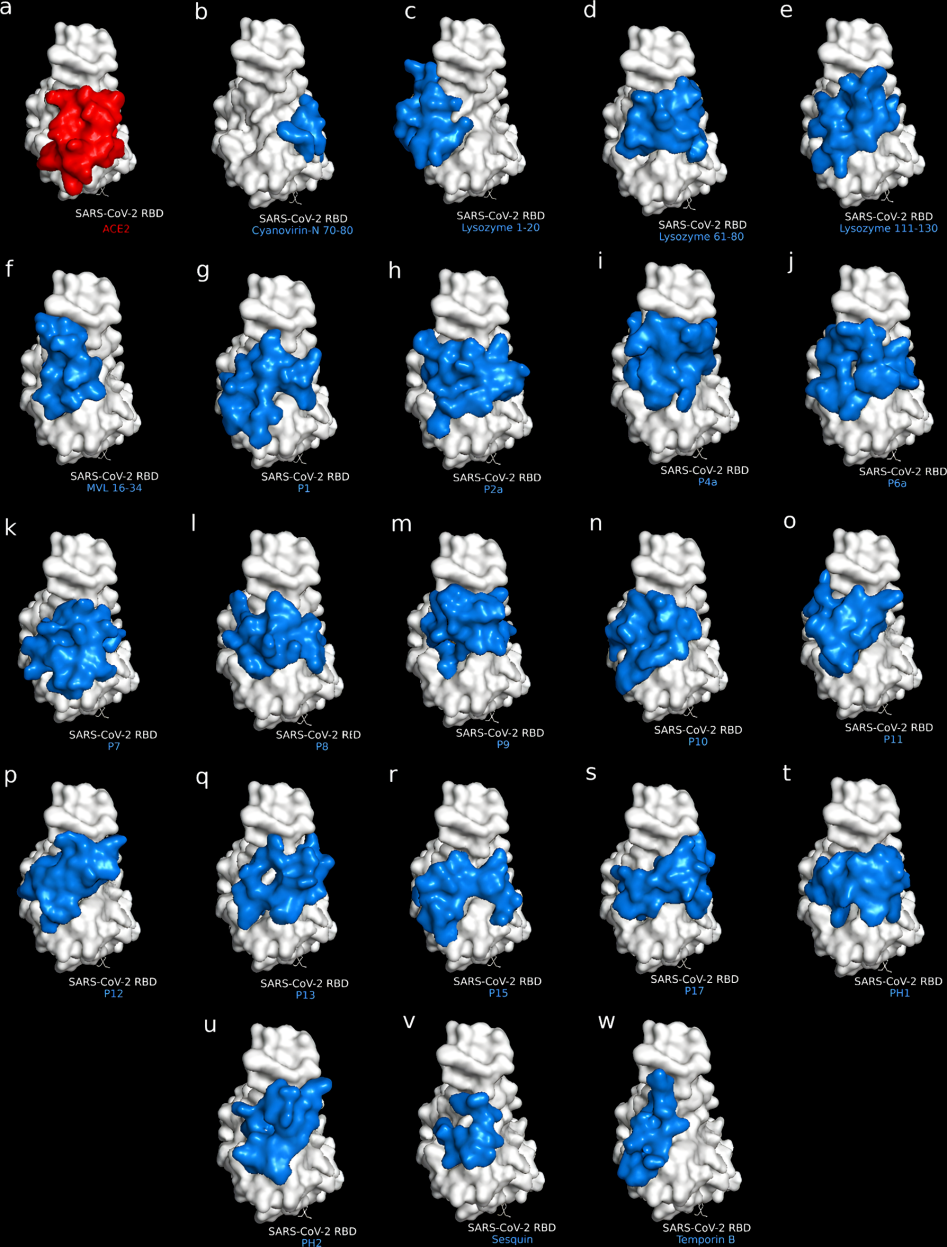
Peptide candidates (blue) docked to the SARS-CoV-2 RBD (white). (a) ACE2 control peptide (red), (b) cyanovirin-N (70–80), (c) lysozyme (1–20), (d) lysozyme (61–80), (e) lysozyme (111–130), (f) MVL (16–34), (g) P1, (h) P2a, (i) P4a, (j) P6a, (k) P7, (l) P8, (m) P9, (n) P10, (o) P11, (p) P12, (q) P13, (r) P15, (s) P17, (t) PH1, (u) PH2, (v) sesquin, and (w) temporin B.

It is important to note that RBD residues from Glu484 to Tyr505, Arg403 to Tyr421, and Tyr449 to Ala475 are involved in the docking of the APD peptides, as was previously reported by Othman and co-workers [6]. Figure 2 shows the mapping of these amino acid residues on the active region of RBD, suggesting that APD peptides are docking on five principal regions of the RBD. The center region of the RBD (Figure S4b and Figure S4c, Supporting Information File 1) is of particular interest because it binds directly to the ACE2 cellular receptor. After this analysis, the number of hydrogen bonds and hydrophobic interactions involved in the APD–RBD complexes was determined by the LigPlot+ software (Table 1) [35]. The numbers of hydrogen bonds per residue and hydrophobic interactions go from 0.25 to 0.95 and from 17 to 31, respectively. The binding affinity values are higher for those APD peptides bound to the RBD with a high number of hydrogen bonds and hydrophobic interactions [47]. Table 1 shows that twelve APD and three lysozyme peptides surpass the binding energy calculated for the ACE2 peptide to the RBD (−4.6 kcal/mol), indicating that these peptides bind to the RBD active region more strongly than the ACE2 peptide.

**Figure 3:**
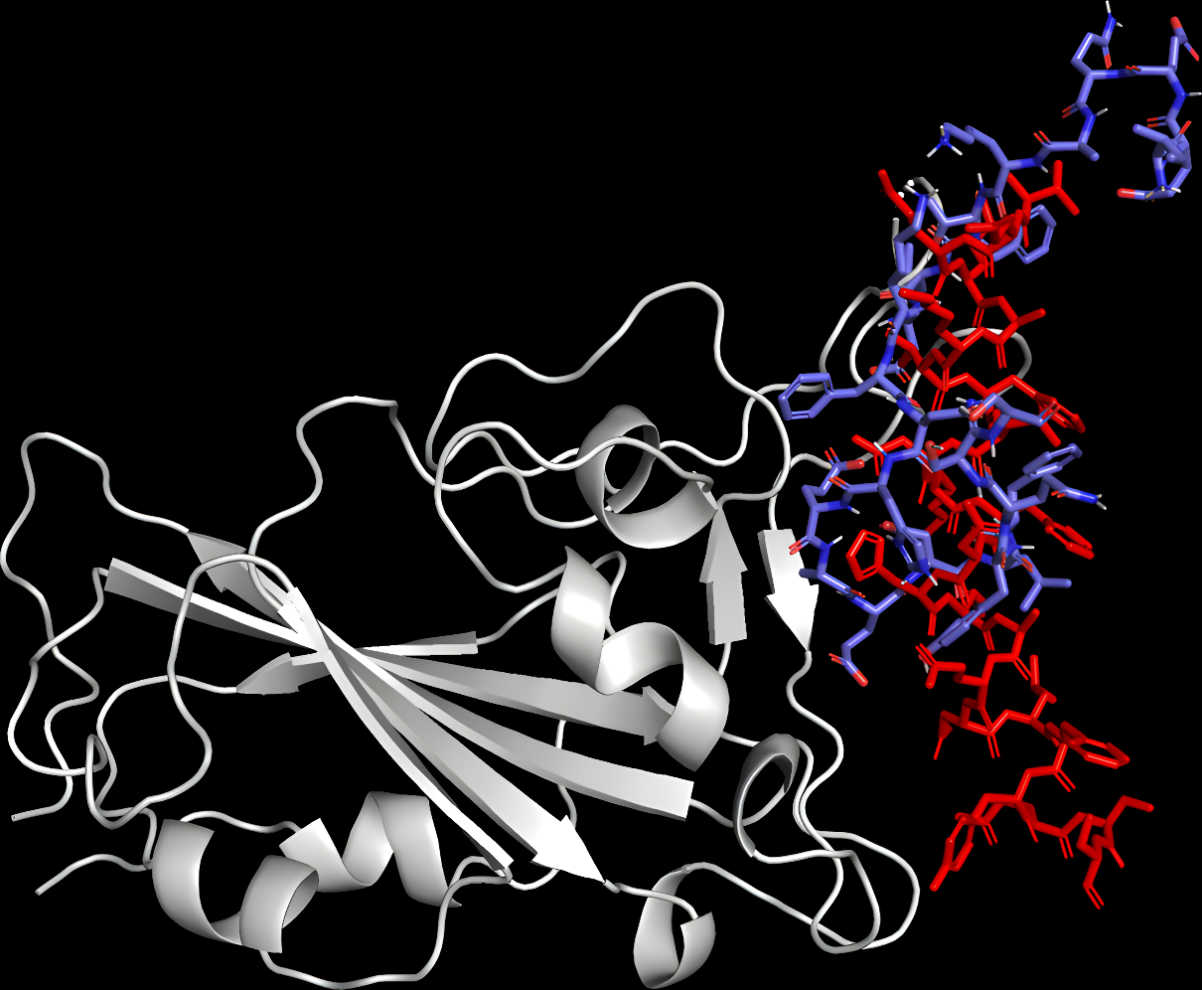
Superimposition of docked ACE2 (blue) onto the crystallized complex (red) in the active site using PyMOL (RMSD = 0.31 Å).

To validate the docking results for the APD peptides, the crystallized ACE2 peptide was tested using the same ADV parameters. The peptide bound on the RBD active region, and superimposing the docked complex onto the crystallized complex showed a low RMSD of 0.31 Å (Figure 3). Generally, an RMSD value of 2 Å or lower is considered a good docking, thus confirming the validity of the protocol [48].

### Proposing new peptides based on hydrogen-bond formation

**Figure 4:**
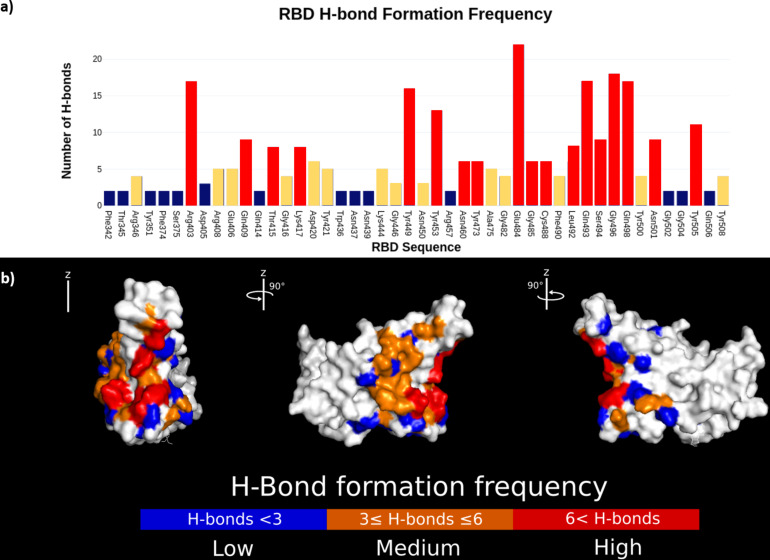
Mapping of the number of hydrogen bonds formed between APD and lysozyme peptide candidates to the SARS-CoV-2 RBD. (a) The graph depicts the residue location and frequency of hydrogen bonds formed with the SARS-CoV-2 RBD. (b) The location of the hydrogen bonds concentrates on the active region of SARS-CoV-2 RBD. The color coding differentiates the frequency of hydrogen bonds formed on each residue with blue being the lowest and red being the highest formation frequency.

Since hydrogen bonds play an important role in the formation and stabilization of the protein–ligand complex, novel peptides were designed considering the most common amino acid residues from the 13 APD and lysozyme peptides that bind to the RBD active region through hydrogen bonds [35]. This task is easy to develop in comparison to the typical procedures used in standard peptide design, in which complex algorithms are used to generate a large peptide library [49–50]. In contrast, designing peptides based on hydrogen bond interactions allows one to generate peptides that target specific sites while reducing computation time [51]. Figure 4 shows the most frequent amino acid residues binding to the RBD active region, together with the implicated amino acid residues in the formation of the RBD–ACE2 complex (Figure 4a) [34]. 41 theoretical peptides (denominated HB peptides) composed of 20 amino acid residues were designed, and from these, 23 HB peptides docked to the RBD.

In agreement with ADV analysis, the HB peptides docked laterally (left and right) and horizontally to the center region of the RBD. Furthermore, the number of hydrogen bonds formed between the amino acid residues from HB peptides and RBD as well as the binding energy between HB peptides and RBD were increased. These results support the success of the chosen strategy for designing peptides based on the hydrogen bond interactions in an easy way.

### Immunogenicity

The immune system can recognize external molecules introduced into our body, which, in many cases, leads to the production of antibodies [12]. Specifically, during a viral infection, viral antigens are presented by MHC I to be recognized by T cells, which, in turn, promote cytosine release and the cytotoxic activity of CD8+ T cells [12,52–53]. Due to the efficiency of this system, many biological therapeutics (proteins, peptides, nucleic acids, and even drugs) do not reach their target since they are eliminated by cells of the immune system, which limits their activity. Therefore, proposed peptides with antiviral activity must be evaluated from the immunological point of view. In this context, an immunogenicity prediction of the proposed peptides (APD, lysozyme, and HB peptides) was developed by the binding of the peptide candidates to MHC I. The final peptide selection was carried out using TepiTool to determine which peptides are capable of evading the immune system, in particular MHC I molecules. The TepiTool platform was used to select peptides of MHC I that do not bind to alleles with an IC_50_ value below 500 nM since, according to Calis and and Adhikari, binding to alleles with an IC_50_ above 500 nM would present low to zero immunogenic response [37–38]. From 55 peptides tested, including APD, lysozyme, and HB peptides, only 22 peptides (Table S4, Supporting Information File 1) had a low probability of being recognized by MHC I. This suggests that these peptides can be used to neutralize the SARS-CoV-2 virus without activating the immune system. These peptides, denominated here “OAPs”, optimally attached to the RBD, and their interaction with the RDB is discussed in the following section.

### Physicochemical parameters and peptide–RBD interaction

Solubility, net charge, and size are important physical parameters that need to be considered in the design of novel drugs since these play a role in the distribution in the human body and in targeting specific cells, bacteria, viruses, or proteins. Therefore, the physicochemical parameters of the peptides and the peptide conformation after binding to RBD were obtained by using WinHydroPro software. The results are given in Table 2. The peptide net charge, isoelectric point, and water solubility for peptides were determined by the on-line software INNOVAGEN’s peptide calculator (PEPCALC). Almost all OAPs are soluble in aqueous media, independently on their isoelectric point, due to the high ratio between hydrophilic and hydrophobic amino acid residues, except the peptides temporin B and lysozyme (61–80). The net charge calculated for the OAPs varies according to the number of negatively and positively charged amino acids present in the primary structure. The net charge values are in the range of −3.9 to 2.9 at pH 7. The OAPs were selected based on an absolute value of the electrical net charge smaller than 3.9 (|charge| ≤ 3.9, which is the net charge of ACE2), to avoid possible cytotoxic effects [54–55].

**Table 2:** Summary of the physicochemical properties of the final peptide candidates.

Peptide	Residues	Number of amino acids	Affinity (kcal/mol)	H bonds/ residue	Water solubility	*R*_g_ (nm)	Net charge at pH 7	Isoelectric point	Molecular weight (kDa)

ACE2	21–44	24	−4.6	0.63	good	1.64	−3.9	4	2890.07
cyanovirin-N	70–80	10	−5.3	0.80	good	0.83	0.9	8.9	1252.40
lysozyme (1–20)	1–20	20	−4.9	0.35	good	0.98	1.9	9.5	2385.81
lysozyme (61–80)	61–80	20	−5.7	0.60	good	1.07	2.9	8.1	2408.69
lysozyme (111–130)	111–130	20	−5.2	0.25	poor	0.83	1.0	9.9	2179.40
MVL (16–34)	16–34	19	−5.8	0.63	good	0.91	0.1	7.9	1938.11
P1	1–20	20	−6.3	0.65	good	0.90	1.0	10	2394.64
P2a	1–20	20	−4.9	0.50	good	0.92	0.1	5.2	2463.58
P4a	1–20	20	−4.8	0.55	good	0.89	0.1	5.2	2463.58
P6a	1–20	20	−4.6	0.55	good	0.86	0.1	5.2	2463.58
P7	1–20	20	−5.0	0.45	good	0.89	0.1	9.5	2463.58
P8	1–20	20	−5.6	0.40	good	0.90	0.1	7.5	2463.58
P9	1–20	20	−5.2	0.50	good	0.87	0.1	7.5	2521.66
P10	1–20	20	−5.5	0.60	good	0.92	−0.9	7.5	2491.59
P11	1–20	20	−5.6	0.55	good	0.86	−0.9	7.5	2491.59
P12	1–20	20	−5.4	0.65	good	0.93	−0.9	7.5	2541.60
P13	1–20	20	−5.2	0.65	good	0.87	1.1	7.5	2532.69
P15	1–20	20	−4.9	0.55	good	0.87	0.1	7.5	2463.58
P17	1–20	20	−4.6	0.40	good	0.91	0.1	7.5	2463.58
PH1	1–20	20	−5.0	0.55	good	0.92	2.0	11.8	2379.51
PH2	1–20	20	−5.3	0.55	good	0.87	0	6.7	2338.41
sesquin	1–10	10	−5.6	0.80	good	0.70	−1.1	3.9	1157.25
temporin B	1–13	13	−5.6	0.69	poor	0.73	1	10.1	1392.77

Given that, at physiological pH, the RBD active region is positively charged, it could be assumed that negatively charged peptides, such as sesquin and MVL (74–87) (Figure S3, panels 4a,b and 14a,b, Supporting Information File 1), would present a stronger binding to the RBD active region than those peptides with slightly positive charge or neutral charge (Figure S3, Supporting Information File 1) due to electrostatic repulsion [56]. However, as seen from the ADV results, cationic peptides, such as lysozyme (61–80) showed a higher binding affinity than the anionic peptides ACE2 and MVL (74–87). The higher binding affinity observed for positively charged peptides can be explained based on the distribution of the electrically charged patches located on the active surface of the RBD. Figure 1b shows the electrostatic surface potential of the RBD active region, in which negatively charged, neutral, and positively charged patches can be identified, depending on the amino acid residues. Therefore, it can be assumed that electrostatic repulsion forces are negligible. This suggests that the intermolecular interactions (hydrogen bonds and hydrophobic interactions) in the APD peptide–RBD complexes are favored. The secondary structure of the APDs changes and adopts a proper conformation to bind to the RBD protein.

**Figure 5:**
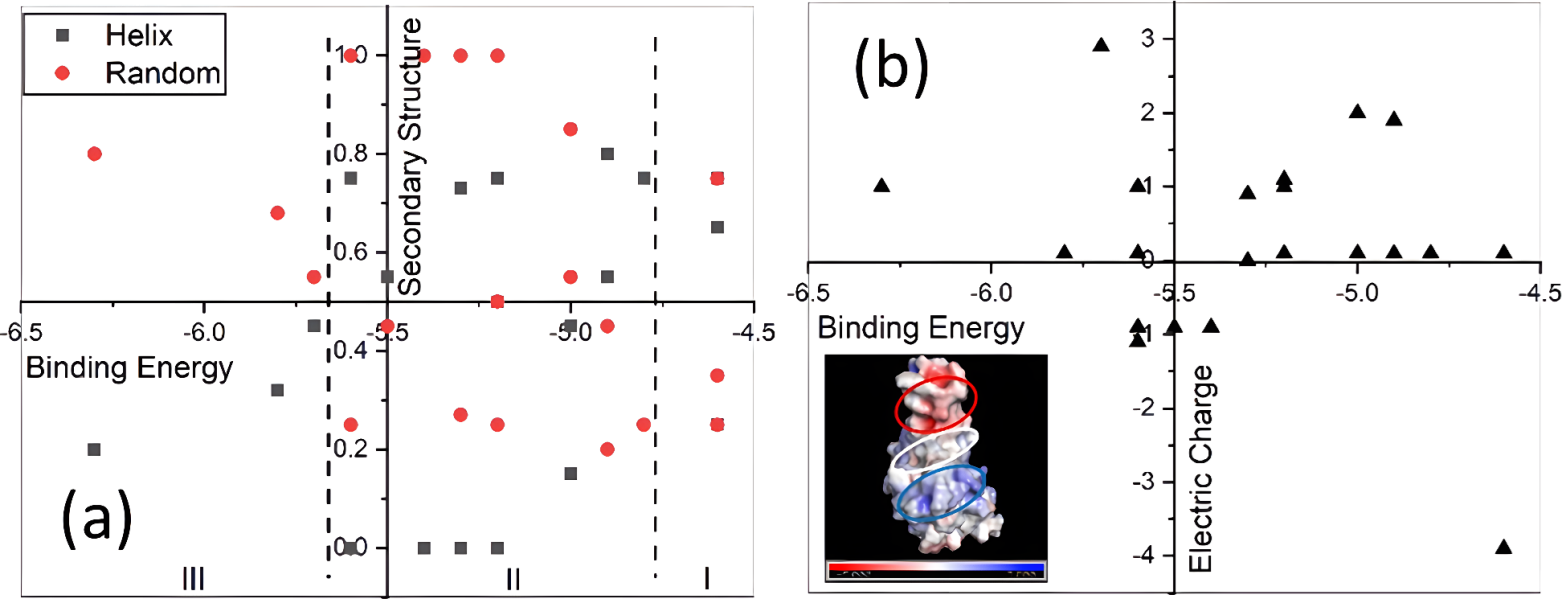
Plots of the peptide secondary structure and charge as functions of the binding energy. (a) Relationship between secondary structure and binding affinity of the peptides to SARS-CoV-2 RBD. (b) Relationship between charge and binding affinity of the peptides to SARS-CoV-2 RBD.

An analysis of the secondary structure for free and docked OAPs was carried out using the PEP-FOLD 3.5 (RPBS Web) web server, while their surface area was analyzed using PyMol. Similar to previous results, the secondary structure of the OAPs changes from α-helices to random-coil conformations, when they docked to the RBD protein, as it is shown in Table 3 [57–59]. The secondary structure for free OAPs consists of α-helices and random coils at different fractions, except for P4a, P6a, temporin B, lysozyme (61–80), PH2, and sesquin, which adopt a fully random-coil conformation. Afterwards, the binding energy of the OAP–RBD complexes, as well as the contact area (*A*_c_) with the RBD active region were determined using docking analysis. The secondary structure and net charge of the OAPs were plotted as functions of the binding energy (Figure 5) with the aim of understanding the relationship between these parameters. Figure 5 shows the secondary structure versus the binding energy of the OAP–RBD complexes (Figure 5a) and is divided into three regions. Region I shows the peptides that have binding energy values similar to the ACE2 binding energy (−4.6 kcal/mol), such as P9 (secondary structure composition 0.75 α-helix and 0.25 random coil) and P12 (fully random-coil conformation). In region II, several peptides present different conformations such as random coils (4), high α-helix-to-random-coil ratio (7), or high-random-coil-to-α-helix ratio (2) conformation. The binding energy in this second region is in the range of −4.8 to −5.6 kcal/mol. The OAPs included in region III are characterized by a high fraction of random-coil secondary structures with binding energies between −5.7 and −6.3 kcal/mol. Additionally, the final surface area (*A*_f_) of peptides docked to the RBD increased, indicated by positive values of ∆*A* = *A*_f_ − *A*_0_, where *A*_f_ and *A*_0_ are the final and initial OAP surface area, respectively (Table 3). In contrast, ACE2 and MVL (16–34) show negative values of ∆*A*. The observed increase of *A*_f_ suggests that the OAPs have a large contact area (*A*_c_) with the active surface area, blocking key amino acid residues involved in the association of RBD with ACE2 (Table 3 and Figure 5), as will be shown next. Figure 5b shows that the binding energy values vary independently of the net charge of the OAPs. This can be explained based on the electrostatic surface potential of the active region of the RBD (inset in Figure 5b), which can be divided into three characteristic regions: (i) The upper region is characterized by a negative potential (red ellipse). (ii) The middle region has neutral patches, slightly negative and positive patches (white ellipse); and (iii) the bottom region is characterized by a positive potential (blue ellipse). Recently, it has been reported that the residues Phe486, Tyr489 (located in the upper region), Gln493, Gly496 (located in the middle region), Thr500, and Asn501 (located in the bottom region), are involved in the association of RBD with the ACE2 protein [6,9]. Interestingly, OAPs were attached in different configurations around the active regions of the RBD (inset in Figure 4b), and those principally occupied the middle region of the active surface*.* These OAPs interact with residues Gln493, Gly496, Thr500, and Asn501, among others amino acidic residues located in the upper and bottom regions. Similar results have been reported by Debmalya and co-workers, who analyzed the potential of chimeric peptides to block the RBD using an in silico approach. They found that a peptide with 26 amino acids binds to the Thr500 and Asn501 residues of the RBD, while a peptide with 23 amino acids binds to the Tyr489 and Thr500 residues of the RBD, and a peptide with 20 amino acid binds to the Gln493 and Asn501 residues of the RBD. However, neither of these peptides was able to block the three regions of the RBD [8]. The results reported herein suggest that OAPs have a great potential as drug inhibitors of SAR-CoV2 and can block the entry of viruses to the cell host through the ACE2 cellular receptor.

**Table 3:** Secondary structure and contact area of peptides. The secondary structure is presented as the number of amino acids in each structure divided by the total number of amino acids of the peptide. *A*_0_ represents the initial surface area, *A*_f_ corresponds to the final surface area, ∆*A* is the change in area (a positive value indicates an increase in area and a negative value indicates a decrease in peptide area), and *A*_c_ is the contact area of the peptide with the SARS-CoV-2 RBD.

OAP	α-Helix	Random coil	*A*_0_ (Å^2^)	*A*_f_ (Å^2^)	Δ*A* (Å^2^)	*A*_c_ (Å^2^)

ACE2	0.25	0.75	3689.47	2544.86	−1144.61	929.92
lysozyme (111–130)	0.45	0.55	2316.07	2695.19	379.12	1113.77
MVL (16–34)	0.32	0.68	1955.31	1938.87	−16.44	449.73
P1	0.20	0.80	2146.66	2789.48	642.82	1092.27
P11	0.75	0.25	2107.56	2635.32	527.76	1078.03
P12	0.65	0.35	1961.34	2527.95	566.61	991.89
P4a	0.00	1.00	1900.02	2438.47	538.45	1019.92
P6a	0.00	1.00	2198.06	2484.03	285.97	962.49
temporin B	0.00	1.00	1444.79	1653.72	208.93	236.14
cyanovirin-N (70–80)	0.73	0.27	1456.50	1472.78	16.28	243.73
lysozyme (1–20)	0.55	0.45	2249.00	2326.36	77.36	907.99
lysozyme (61–80)	0.00	1.00	1737.46	2493.78	756.32	1021.43
P10	0.55	0.45	2104.89	2635.63	530.74	1031.17
P13	0.45	0.55	2071.74	2706.08	634.34	1046.71
P15	0.75	0.25	2064.25	2774.07	709.82	1139.50
P17	0.50	0.50	2035.18	2684.64	649.46	1053.52
P2a	0.55	0.45	2024.86	2416.36	391.50	940.54
P7	0.75	0.25	1963.84	2914.81	950.97	1113.68
P8	0.80	0.20	1935.11	2645.75	710.64	993.20
P9	0.75	0.25	2176.42	2592.66	416.24	983.47
PH1	0.15	0.85	2008.82	2483.21	474.39	1f007.82
PH2	0.00	1.00	2030.17	2509.95	479.78	987.57
sesquin	0.00	1.00	1212.56	1454.82	242.26	132.90

### Binding energy by protein binding energy prediction

**Figure 6:**
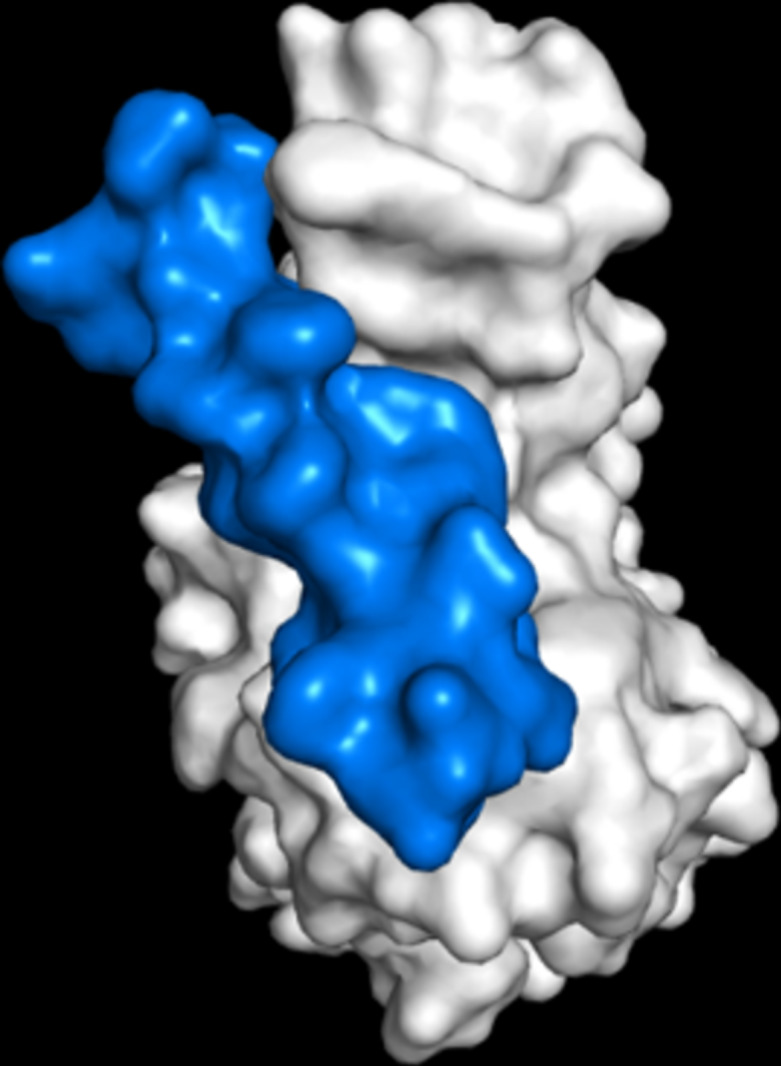
Crystallized ACE2 peptide (I21 to S44) bound to the SARS-CoV-2 RBD.

ADV has been widely used to predict the alignment of small ligands within the binding cavity of a given protein and to evaluate the pose quality of the docked ligand in terms of binding energy. However, this computational tool gives comparably low binding energies of peptide–protein docking due to the molecular size, high flexibility, and complexing conformation of the peptide ligand, in addition to the simplification of the analysis of ADV (the electrostatic and solvation potentials are neglected, while van der Waals potential, the nondirectional hydrogen bond term, the hydrophobic term, and a conformational entropy penalty are considered) [20–21]. It can be observed that the binding energy values (Table 1 and Table 2) are significantly lower than the binding energies reported in similar works [6,60–61]. For instance, the experimental and theoretical values of the binding energy reported for ACE2–RBD is around −12.0 kcal/mol, which is higher than the binding energy of −4.6 kcal/mol given here [61]. Therefore, the PRODIGY web server was used to estimate the binding energy for peptide–RBD docking, since it has been demonstrated that PRODIGY can produce results comparable with those obtained experimentally and most standard in silico analyses [38–40,60]. To validate the PRODIGY results, the binding energy of the complex ACE2–RBD (6VYB) (Figure 6), acquired from the RCSB Protein Data Bank (https://www.rcsb.org/), was first tested. This value is similar to the binding energy previously reported for the crystalline complex [61].

**Figure 7:**
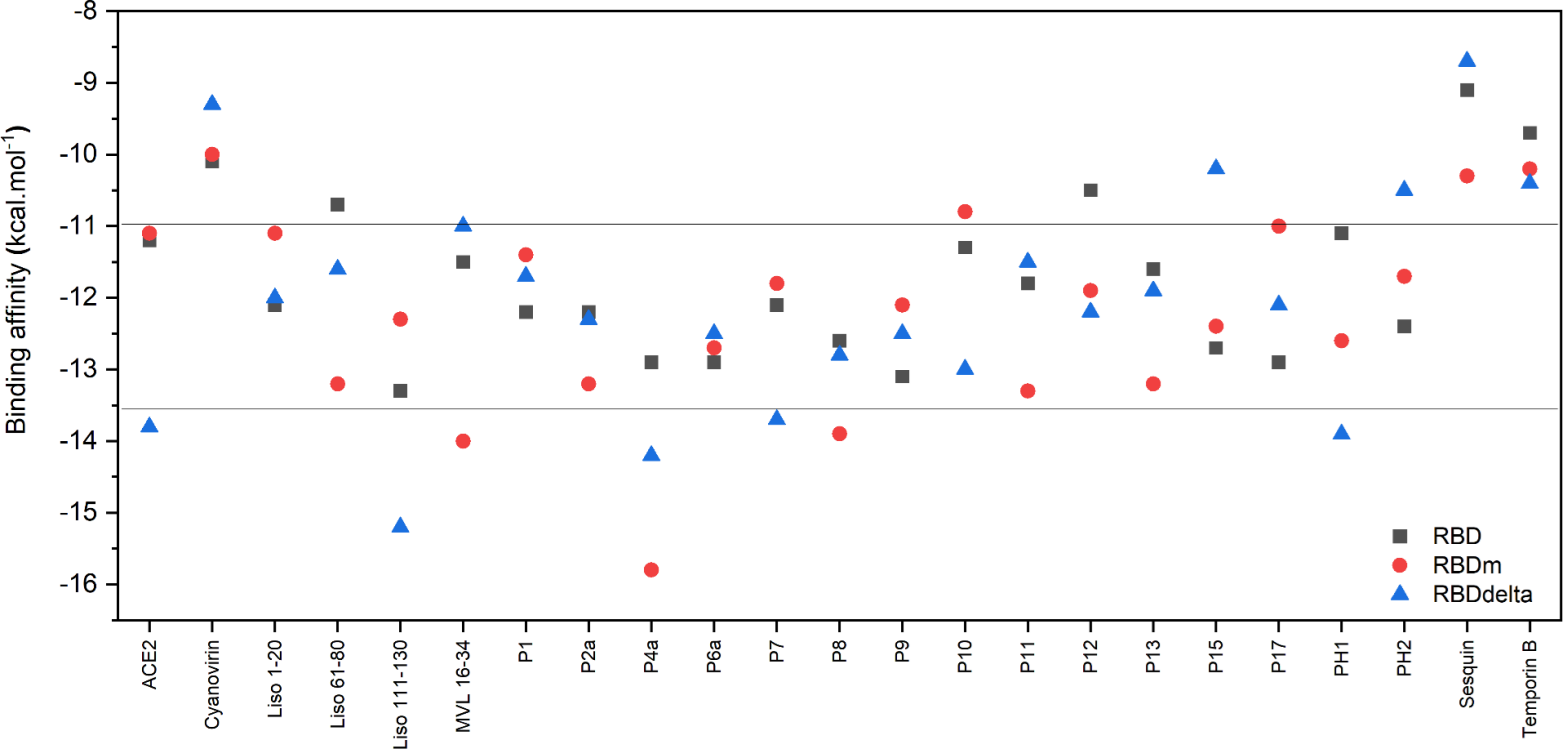
Binding affinity calculated using the PRODIGY server from ADV results. The binding affinities were calculated for different RBD variants: Wuhan strain RBD (black), RBDm (red), RBDδ (blue).

The binding energy values for the OAP–RBD complexes vary from −9.2 kcal/mol to −13.3 kcal/mol (Figure 7). These results are similar to previous reports in which short peptides were docked to RBD [14]. However, the binding energy reported in the present research contrasts with several studies that reported higher binding energies between the theoretical peptides and RBD [62–63], probably due to the software used in the molecular docking calculations. However, these reports did not show the binding energy of the ACE2–RBD complex. 15 OAPs surpass the energy value registered for ACE2–RBD. It is important to recall that OAPs are attached to critical RBD amino acid residues (Phe486, Tyr489, Gln493, Gly496, Thr500, and Asn501) involved in the entry of SARS-CoV-2 into the host cell. These results indicate a possible application of these peptides in the prophylaxis of COVID-19 disease caused by different variants of SARS-CoV-2. In this regard, OAPs were also docked against two variants of RBD: a theoretical multimutation variant (RBDm), which encompasses the mutations found in Alpha, Beta, and Gamma SARS-CoV-2 variants, and the Delta variant (RBDδ).

The binding affinities of the OAPs with each RBD variant are shown in Figure 7. The binding affinities of the OAPs vary according to the RBD variant. The binding affinity of sixteen OAPs is shown to be higher in comparison to ACE2 for RBDm, highlighting MVL (16–34), P2a, and P8 for their higher binding affinity to the RBDm. Four OAPs (lysozyme (111–130), P4a, P7, and PH1) showed a higher binding affinity to RBDδ than the ACE2 peptide, where the binding affinity of lysozyme (111–130) stands out compared to that of other OAPs. It important to note that lysozyme (111–130) and P4a consistently present a stronger binding affinity for all RBD variants than ACE2, making them the best candidates against all RBD variants.

Last, the radius of gyration of these peptides was determined to be in the range of 43% to 65% of the size of the ACE2 peptide. Therefore, the diffusion of these peptides is faster. These results show the potential of the selected peptides to inhibit SARS-CoV-2, considering that their smaller size and faster diffusion will allow them to find the virus faster and bind to the RBD, thus blocking its interaction with the ACE2 receptor.

## Conclusion

Molecular docking was used to analyze the interaction of 104 peptides from the APD, recognized by their antimicrobial and antiviral activity, with the RBD of SARS-CoV-2. This analysis allowed for the selection (16 peptides) and faster design of peptides (41 peptides) based on the peptide binding site on the RBD, the number of hydrogen bonds, and the binding affinity. The peptide candidates have a nearly neutral charge at physiological pH and good solubility, which can benefit the diffusion of the molecules, allowing them to reach and efficiently bind to the RBD active region. After the immunogenicity analysis with MHC I, 22 peptides (15 theoretical peptides) were chosen because of their potential capability to inhibit the RBD of SARS-CoV-2. Since they interact with F486, Y489, Q493, G496, T500, and N501 residues present on the RBD, they play an important role in the infection mechanism of SARS-CoV-2. Also, these peptides showed a higher binding affinity for different RBD variants (Delta variant and a theoretical multimutation variant obtained from the combination of Alpha, Beta and Gamma SARS-CoV-2 variants), suggesting their potential use as therapeutic agents against COVID-19. Despite the fact that ADV analysis is a powerful tool, additional experimental and in silico assays are required to determine the preference of OAP peptides for binding to the RBD protein, instead of binding to other viral proteins or common proteins (salivary and plasmatic proteins) found in physiological fluids. The procedure described here for the design of antiviral molecules can be extended against other viruses, both in human and veterinary medicine.

## Supporting Information

Supporting Information features previously reported antiviral activities of APD peptides (Table S1); molecular docking scores of the 104 peptides (Table S2); Ligplot+ diagrams of the hydrogen bonds and hydrophobic interactions between ACE2 and the SARS-CoV-2 RBD (Figure S1); distribution of electrostatic potential on the surface of APD peptide candidates docked to the SARS-CoV-2 RBD (Figure S2); secondary structure, docking, and distribution of electrostatic charges of aligned peptides (Figure S3); physicochemical properties, hydrogen bonds, and hydrophobic interactions of the peptide candidates against SARS-CoV-2 (Table S3); principal docking regions of screened APD and lysozyme peptides against the SARS-CoV-2 RBD (Figure S4); immunogenicity analysis of peptides against human MHC I to determine the number of alleles with IC_50_ < 50 nM and IC_50_ < 500 nM (Table S4); and the contact areas of peptides docked to SARS-CoV-2.

File 1 Additional experimental data.
