# Supplementary material for: Design and selection of peptides to block the SARS-CoV-2 receptor binding domain by molecular docking
Source: Beilstein J Nanotechnol. 2022 Jul 22;13:699–711. doi: 10.3762/bjnano.13.62 (PMC9344557; doi:10.3762/bjnano.13.62)
Supplement: File 1 — Additional experimental data. [file Beilstein_J_Nanotechnol-13-699-s001.pdf]

BEILSTEIN JOURNAL OF NANOTECHNOLOGY

Supporting Information

for

Design and selection of peptides to block the SARS-CoV-2 receptor binding domain by molecular docking

Kendra Ramirez-Acosta, Ivan A. Rosales-Fuerte, J. Eduardo Perez-Sanchez, Alfredo Nuñez-Rivera, Josue Juarez and Ruben D. Cadena-Nava

Beilstein J. Nanotechnol. **2022,** *13,* 699–711. doi:10.3762/bjnano.13.62

Additional experimental data

License and Terms: This is a supporting information file under the terms of the Creative Commons Attribution License (https://creativecommons.org/licenses/by/4.0). Please note that the reuse, redistribution and reproduction in particular requires that the author(s) and source are credited and that individual graphics may be subject to special legal provisions.
The license is subject to the *Beilstein Journal of Nanotechnology* terms and conditions: (https://www.beilstein-journals.org/bjnano/terms)

Table S1: Summary of antiviral peptides used for initial docking; their known antiviral activity is listed. Information taken from the Antimicrobial Peptide Database (https://aps.unmc.edu/database/anti).

APD ID	PEPTIDE NAME	ACTIVITY
**AP00013**	Aurein 1.2	Anti-HIV
**AP00023**	Antiviral protein Y3	Inhibition of Tobacco Mosaic Virus (TMV)
**AP00025**	Alloferon 1	Inhibition of Herpes Simplex Virus (HSV)
**AP00026**	Lactoferricin B	Anti-HIV
**AP00028**	Tricyclic peptide RP 71955	Anti-HIV
**AP00029**	Cecropin A(1-8)-Magainin 2(4-12) hybrid peptide (CE-MA)	Anti-HIV
**AP00058**	Maximin 1	Anti-HIV
**AP00060**	Maximin 3	Anti-HIV
**AP00061**	Maximim 4	Anti-HIV
**AP00062**	Maximin 5	Anti-HIV
**AP00074**	Brevinin-1	Inhibition of Herpes Simplex Virus (HSV)
**AP00094**	Temporin A	Active against Channel Catfish Virus (CCV) and Frog Virus 3 (FV3)
**AP00095**	Temporin B	Inhibition of Herpes Simplex Virus (HSV)
**AP00102**	Thanatin	Inhibition of Tobacco Mosaic Virus (TMV)
**AP00121**	RANATUERIN 2P	Active against Channel Catfish Virus (CCV) and Frog Virus 3 (FV3)
**AP00139**	Cecropin A	Anti-HIV
**AP00144**	Magainin 2	Anti-HIV
**AP00146**	Melittin	Inhibition of Herpes Simplex Virus (HSV-1 & HSV-2)
**AP00150**	Indolicidin	Anti-HIV, Anti-HSV-1, Anti-HSV-2
**AP00160**	Dermaseptin-S4	Anti-HIV
**AP00173**	GNCP-2	Neutrophil. Generic antiviral activity is inferred.
**AP00174**	GNCP-1	Neutrophil. Generic antiviral activity is inferred.
**AP00176**	human neutrophil peptide-1	Anti-HIV. Active against Zika Virus.
**AP00177**	human neutrophil peptide-2	Inhibition of Herpes Simplex Virus (HSV-1 & HSV-2)
**AP00178**	human neutrophil peptide-3	Anti-HIV. Active against pseudo typed viruses expressing SARS-CoV-2 spike proteins.
**AP00179**	human neutrophil peptide-4	Anti-HIV
**AP00180**	human defensin 5	Inhibits non-enveloped BK virus infection.
**AP00181**	human defensin 6	Human defensin. Innate antiviral activity is expected.
**AP00187**	Rabbit neutrophil peptide 1	Neutrophil. Generic antiviral activity is inferred.
**AP00188**	Rabbit neutrophil defensin 2	Neutrophil. Generic antiviral activity is inferred.
**AP00195**	Protegrin 1	Anti-HIV
**AP00211**	Polyphemusin I	Anti-HIV
**AP00212**	Polyphemusin II	Anti-HIV
**AP00214**	Tachyplesin I	Anti-HIV, Anti-HSV-1, Anti-HSV-2
**AP00217**	Rabbit neutrophil defensin 3a	Neutrophil. Generic antiviral activity is inferred.
**AP00218**	Protegrin 2	Anti-HIV
**AP00219**	Protegrin 3	Anti-HIV
**AP00220**	Protegrin 4	Anti-HIV
**AP00221**	Protegrin 5	Anti-HIV
**AP00222**	RatNP-1	Neutrophil. Generic antiviral activity is inferred.
**AP00223**	RatNP-2	Neutrophil. Generic antiviral activity is inferred.
**AP00224**	RatNP-3	Neutrophil. Generic antiviral activity is inferred.

**AP00225**	RatNP-4	Neutrophil. Generic antiviral activity is inferred.
**AP00240**	Caerin 1.1	Anti-HIV
**AP00241**	Caerin 1.2	Anti-HIV
**AP00242**	Caerin 1.3	Anti-HIV
**AP00243**	Caerin 1.4	Anti-HIV
**AP00244**	Caerin 1.5	Anti-HIV
**AP00245**	Caerin 1.6	Anti-HIV
**AP00246**	Caerin 1.7	Anti-HIV
**AP00257**	Caerin 4.1	Anti-HIV
**AP00272**	mBD-1	A fusion construction of mBD-1 and mBD-3 is active against Influenza A Virus (IAV).
**AP00274**	Circulin A	Anti-HIV
**AP00275**	Circulin B	Anti-HIV
**AP00281**	mCRAMP	Inactivates Zika virus (ZIKV).
**AP00283**	Human beta defensin 3	Anti-HIV
**AP00310**	LL-37	Anti-HIV, Anti-HSV-1, Anti-HSV-2. Active against Zika virus and Dengue virus type 2.
**AP00325**	Uperin 3.6	Anti-HIV
**AP00327**	Uperin 7.1	Anti-HIV
**AP00333**	Mytilin B	Related to innate antiviral immune response.
**AP00345**	Caerin 1.10	Anti-HIV
**AP00355**	Ginkbilobin	Anti-HIV
**AP00366**	BMAP-27	Anti-HIV
**AP00367**	BMAP-28	Anti-HSV-1
**AP00384**	Ponericin L2	Anti-HIV
**AP00399**	Spinigerin	Anti-HIV
**AP00405**	RANATUERIN 6	Anti-HIV
**AP00408**	RANATUERIN 9	Anti-HIV
**AP00445**	RTD-1	Active against HIV and SARS virus.
**AP00446**	Alpha-basrubrin	Anti-HIV
**AP00449**	Alpha-MSH	Anti-HIV
**AP00451**	hBD-1	Anti-HIV
**AP00473**	Piscidin 1	Anti-HIV
**AP00474**	Piscidin 3	Active against Channel Catfish Virus (CCV).
**AP00499**	Gramicidin A	Anti-HIV
**AP00505**	human Histatin 5	Anti-HIV
**AP00524**	Human beta defensin 2 (monomero)	Anti-HIV
**AP00549**	Plectasin	Active against dengue virus type 2.
**AP00553**	Sesquin	Anti-HIV
**AP00708**	GF-17	Anti-HIV
**AP00729**	Kalata B1	Anti-HIV
**AP00730**	Kalata B8	Anti-HIV
**AP00928**	Subtilosin A	Active against HSV-1 and HSV-2.
**AP01010**	Latarcin 1	Active against dengue virus type 2.
**AP01034**	Palicourein	Anti-HIV
**AP01049**	Kalata B2	Anti-HIV
**AP01058**	Vhl-1	Anti-HIV

**AP01065**	Cycloviolacin O14	Anti-HIV
**AP01136**	Tricyclon A	Anti-HIV
**AP01208**	Retrocyclin-2	Anti-HIV
**AP01382**	TEWP	Active against Chandipura virus.
**AP01406**	EP5-1	Active against pseudorabies virus (PRV).
**AP01580**	Elafin	Anti-HIV, Anti-HSV-1, Anti-HSV-2
**AP01654**	Caerin 1.19	Anti-HIV
**AP02095**	SLPI	Anti-HIV
**AP02099**	RNase 3	Active against respiratory syncytial virus, group B (RSV-B).
**AP02130**	Antiviral lectin scytovirin	Anti-HIV
**AP02131**	Cyanovirin-N	Anti-HIV
**AP02132**	Microcystis viridis lectin	Anti-HIV
**AP02133**	Griffithsin	Anti-HIV
**AP02146**	ALFpm3	Active against White Spot Syndrome Virus (WSSV).
**AP02337**	RNase 2	Active against respiratory syncytial virus (RSV).

Table S2: Molecular docking scores of the 104 peptides using AutoDock Vina. The table shows the biochemical properties and the peptide's organism of origin.

Peptide	Sequence fragment	PDB/ *UNIPRO*	Residues	Sequence	Affinity (kcal/mol)	H bonds/ Residues	Organism of origin
**Alpha-Basrubrin**	1-20	P83186	20	GADFQECMKEHSQKQHQHQG	-5.2	0.95	*Basella alba*
**Human Beta Defensin 3**	27-45	1KJ6	19	EEQIGKCSTRGRKCCRRKK	-5.0	0.95	*Homo sapiens*
**Sesquin**	1-10	P84868	10	KTCENLADTY	-5.6	0.80	*Vigna unguiculata subsp. sesquipedalis*
**Indolicidin**	1-13	1G89	13	ILPWKWPWWPWRR	-8.0	0.77	*N/A*
**BMAP-28**	1-18	2NDC	18	GGLRSLGRKILRAWKKYGPIIVPIIRIG	-4.4	0.77	*Bos taurus*
**GF-17**	1-17	2L5M	17	GFKRIVQRIKDFLRNLV	-5.3	0.76	*synthetic construct*
**Cyanovirin-N (70-80)**	70-80	2EZM	10	AAECKTRAQQ	-5.3	0.73	*Nostoc ellipsosporum*
**Protegrin 5**	1-18	2NC7	18	RGGRLCYCRPRFCVCVGR	-7.2	0.72	*Sus scrofa (Porcine)*
**MVL (94-110)**	94-110	1ZHS	17	GQWRTIVEGVMSVIQIK	-5.0	0.71	*Microcystis viridis*
**Temporin B**	1-13	6GIL	13	LLPIVGNLLKSLL	-5.6	0.69	*Rana temporaria*
**Dermaseptin-S4**	1-13	2DD6	13	ALWKTLLKKVLKA	-5.5	0.69	*N/A*
**MVL (74-87)**	74-87	1ZHS	14	NDEAQKLGPQIAAS	-5.9	0.64	*Microcystis viridis*
**MVL (16-34)**	16-34	1ZHS	19	AEAQQVGPKIAAAHQGNFT	-5.8	0.63	*Microcystis viridis*
**ACE2**	21-44	6VYB	24	IEEQAKTFLDKFNHEAEDLFYQSS	-4.6	0.63	*Homo sapiens*
**RTD-1**	1-18	2LYF	18	GFCRCLCRRGVCRCICTR	-7.1	0.61	*N/A*
**Spinigerin**	1-25	1ZRV	25	HVDKKVADKVLLLKQLRIMRLLTRL	-5.0	0.6	*N/A*
**RNase 3**	33-42	2BZZ	10	NYQRRCKNQN	-6.0	0.6	*Homo sapiens*
**Protegrin 1**	1-18	1PG1	18	RGGRLCYCRRRFCVCVGR	-6.9	0.56	*Sus scrofa*
**LL-37 core**	17-29	2K6O	13	FKRIVQRIKDFLR	-4.8	0.54	*Homo sapiens*
**Latarcin 1**	1-26	2PCO	26	SMWSGMWRRKLKKLRNALKKKLKGEK	-4.5	0.54	*N/A*
**Gramicidin A**	1-15	1MAG	15	VGALAVVVWLWLWLW	-6.4	0.53	*Brevibacillus brevis*
**MVL (64-82)**	64-82	1ZHS	19	TDVLAGPLWSNDEAQKLGP	-5.0	0.53	*Microcystis viridis*
**Piscidin 1**	1-22	2JOS	22	FFHHIFRGIVHVGKTIHRLVTG	-5.3	0.5	*N/A*
**Human Histatin 5**	1-24	P15516	24	DSHAKRHHGYKRKFHEKHHSHRGY	-4.4	0.5	*Homo sapiens*
**ALC (51-90)**	51-90	2QT4	40	GPTYCWDEAKNPGGPNRCSNSKQCDGARTCSSSGFCQGTA	-5.1	0.5	*Scytonema varium*
**MVL (35-42)**	35-42	1ZHS	8	GQWTTVVE	-6.1	0.5	*Microcystis viridis*
**MVL (60-74)**	60-74	1ZHS	15	HEFKTDVLAGPLWSN	-4.6	0.47	*Microcystis viridis*
**Tachyplesin 1**	1-17	1WO1	17	KWCFRVCYRGICYRRCR	-5.9	0.47	*Tachypleus tridentatu*
**Alpha-MSH**	1-13	P01190	13	SYSMEHFRWGKP	-5.7	0.46	*Bos taurus*
**MVL (10-30)**	10-30	1ZHS	20	GPLWSNAEAQQVGPKIAAAH	-6.1	0.45	*Microcystis viridis*
**MVL (42-57)**	42-57	1ZHS	16	ESAMSVVEVELQVENT	-5.2	0.44	*Microcystis viridis*
**Protegrin 2**	1-16	2MUH	16	RGGRLCYCRRRFCVCV	-6.5	0.44	*Sus scrofa*
**Protegrin 4**	1-18	6QKF	18	RGGRLCYCRGWICFCVGR	-7.0	0.44	*Sus scrofa*
**Ranatuerin-9**	1-14	P82824	14	FLFPLITSFLSKVL	-5.8	0.43	*Lithobates catesbeianus*

**Caerin 1.7**	1-24	P62548	24	GLFKVLGSVAKHLLPHVAPVIAEK	-5.5	0.42	*Ranoidea xanthomera*
**BMAP-27**	1-27	2KET	27	GRFKRFRKKFKKLFKKLSPVIPLLHLG	-4.0	0.41	*Bos taurus*
**Caerin 1.5**	1-25	P56230	25	GLLSVLGSVVKHVIPHVVPVIAEHL	-4.3	0.4	*Ranoidea caerulea*
**Retrocyclin-2**	1-18	2LZI	18	GICRCICGRRICRCICGR	-7.4	0.39	*N/A*
**Caerin 1.6**	1-24	P62546	24	GLFSVLGAVAKHVLPHVVPVIAEK	-5.4	0.38	*Ranoidea xanthomera*
**Piscidin 3**	1-22	2MCX	22	FIHHIFRGIVHAGRSIGRFLTG	-5.7	0.36	*Morone chrysops*
**Caerin 4.1**	1-23	P56242	23	GLWQKIKSAAGDLASGIVEGIKS	-4.1	0.35	*Ranoidea caerulea*
**Uperin 3.6**	1-17	P82050	17	GVGDLIRKAVSVIKNIV	-5.0	0.35	*Litoria ewingii*
**GNCP-2**	1-32	P49112	32	GRRCICTTRTCRFPYRRLGTCLFQNRVYTFCC	-5.5	0.34	*Cavia porcellus*
**NCP 2**	61-93	P49112	32	GRRCICTTRTCRFPYRRLGTCLFQNRVYTFCC	-5.5	0.34	*Cavia porcellus*
**RNase 3 (beta)**	38-46	1H1H	9	CKNQNTFLR	-10.8	0.33	*Homo sapiens*
**Griffithsin**	19-36	2GTY	18	LSSIAVRLYSGSDAIIID	-5.3	0.33	*Griffithsia*
**Caerin 1.1**	1-25	P62568	25	GLLSVLGSVAKHVLPHVVPVIAEHL	-5.1	0.32	*Ranoidea splendida*
**Caerin 1.19.3**	7-25	P0C2A8	19	GSVAKHLLPHVAPIIAEKL	-5.6	0.32	*Ranoidea gracilenta*
**Rabbit neutrophil peptide 1**	1-31	P07469	31	ICACRRRFCPNSERFSGYCRVNGARYVRCCS	-4.4	0.32	*Oryctolagus cuniculus*
**Corticostatin 1**	61-91	P07469	31	ICACRRRFCPNSERFSGYCRVNGARYVRCCS	-4.4	0.32	*Oryctolagus cuniculus*
**Uperin 7.1**	1-13	P82050	13	GWFDVVKHIASAV	-5.6	0.31	*Litoria ewingii*
**Ranatuerin-6**	1-13	P82821	13	FISAIASMLGKFL	-6.6	0.31	*Lithobates catesbeianus*
**Rabbit neutrophil defensin 2**	1-32	P07468	32	RCVCRKQLLCSYRERRIGDCKIRGVRFPFCCP	-4.4	0.31	*Oryctolagus cuniculus*
**Corticostatin 2**	2-33	P07468	32	RCVCRKQLLCSYRERRIGDCKIRGVRFPFCCP	-4.4	0.31	*Oryctolagus cuniculus*
**Ginkbilobin**	1-40	P83171	40	ANTAFVSSAHNTQKIPAGAPFNRNLRAMLADLRQNAAFAG	-3.1	0.28	*Ginkgo biloba*
**Caerin 1.4**	1-25	P62544	25	GLLSSLSSVAKHVLPHVVPVIAEHL	-5.7	0.28	*Ranoidea gilleni*
**ALC (3-42)**	3-42	2QT4	40	GPTYCWNEANNGPGPNRCCSSSGFCCQKNNSDGARTQGTS	-4.7	0.28	*Scytonema varium*
**Polyphemusin 1**	1-18	1RKK	18	RRWCFRVCYRGFCYRKCR	-5.5	0.28	*Synthetic construct*
**Protegrin 3**	1-18	2MZ6	18	RGGGLCYCRRRFCVCVGR	-6.2	0.28	*Sus scrofa*
**mCRAMP**	1-34	P51473	34	GLLRKGGEKIGEKLKKIGQKIKNFFQKLVPQPEQ	-3.5	0.26	*Xenopus laevis*
**RatNP-2**	1-31	Q62715	31	TCYCRSTRCGFRERLSGACGYRGRIYRLCCR	-3.9	0.26	*Rattus norvegicus*
**RatNP-4**	1-31	Q62714	31	ACYCRIGACVSGERLTGACGLNGRIYRLCCR	-4.3	0.26	*Rattus norvegicus*
**RNase 3 (1-19)**	1-19	1H1H	19	RPPQFTRAQWFAIQHISLN	-10.1	0.26	*Homo sapiens*
**Caerin 1.10**	1-25	P82104	25	GLLSVLGSVAKHVLPHVVPVIAEKL	-4.2	0.24	*Ranoidea splendida*
**Caerin 1.2**	1-25	P56227	25	GLLGVLGSVAKHVLPHVVPVIAEHL	-4.8	0.24	*Ranoidea caerulea*
**Caerin 1.3**	1-25	P56228	25	GLLSVLGSVAQHVLPHVVPVIAEHL	-4.7	0.24	*Ranoidea caerulea*
**Cyanovirin-N (40-56)**	40-56	2EZM	17	IENVDGSLKWQPSNFIE	-5.4	0.24	*Nostoc ellipsosporum*
**GNCP-1**	1-30	Q64365	30	RCICTTRTCRFPYRRLGTCIFQNRVYTFCC	-4.7	0.23	*Cavia porcellus*
**NCP 1 type B**	64-93	Q64365	30	RCICTTRTCRFPYRRLGTCIFQNRVYTFCC	-4.7	0.23	*Cavia porcellus*

**Human Beta Defensin 1**	33-68	1E4S	36	DHYNCVSSGGQCLYSACPIFTKIQGTCYRGKAKCC	-4.3	0.22	*Homo sapiens*
**Human neutrophil peptide-2**	1-28	1ZMI	28	CYCRIPACIAGERRYTCIYQGRLWAFCC	-4.8	0.21	*Homo sapiens*
**Neutrophil defensin 2**	1-28	1ZMI	28	CYCRIPACIAGERRYTCIYQGRLWAFCC	-4.8	0.21	*Homo sapiens*
**Plectasin**	1-40	3E7R	40	GFGCNGPWDEDDMQCHNHCKSIKGYKGGYCAKGGFVCKCY	-3.8	0.2	*N/A*
**EP5-1**	1-5	P84182	5	ACSAG	-4.5	0.20	*Eisenia fetida*
**Caerin 1.19**	1-25	P0C2A8	25	GLFKVLGSVAKHLLPHVAPIIAEKL	-4.3	0.20	*Ranoidea gracilenta*
**SLPI**	1-50	2Z7F	50	RRKPGKCPVTYGQCCGKSCCQGDMECCKLDRKCMGMFNPPNLMLVSPVKA	-4.3	0.20	*Homo sapiens*
**Human neutrophil peptide-3**	1-30	1DFN	30	DCYCRIPACIAGERRYGTCIYQGRLWAFCC	-5.1	0.20	*Homo sapiens*
**Defensin HNP-3**	1-30	1DFN	30	DCYCRIPACIAGERRYGTCIYQGRLWAFCC	-5.1	0.20	*Homo sapiens*
**Circulin B**	1-31	2ERI	31	CGESCVFIPCISTLLGCSCKNKVCYRNGVIP	-4.3	0.19	*Chassalia parviflora*
**TEWP**	1-36	2B5B	36	EKKCPGRCTLKCGKHERPTLPYNCGKYICCVPVKVK	-4.3	0.19	*Caretta caretta*
**Human defensin 5**	1-32	1ZMP	32	ATCYCRTGRCATRESLSGVCEISGRLYRLCCR	-3.7	0.19	*Homo sapiens*
**Human defensin 6**	1-32	3QTE	32	AFTCHCRRSCYSTEYSYGTCTVMGINWRFCCL	-8.6	0.19	*Homo sapiens*
**Rabbit neutrophil defensin 3**	1-32	1EWS	32	MPCSCKKYCDPWEVIDGSCGLFNSKYICCREK	-4.5	0.19	*Oryctolagus cuniculus*
**hAD 6 (H27W mutant)**	1-32	3QTE	32	AFTCHCRRSCYSTEYSYGTCTVMGINWRFCCL	-4.3	0.19	*Homo sapiens*
**Mytilin B**	1-34	2EEM	34	SCASRCKGHCRARRCGYYVSVLYRGRCYCKCLRC	-4.3	0.18	*N/A*
**Human Beta Defensin 2**	28-64	1E4Q	37	PVTCLKSGAICHPVFCPRRYKQIGTCGLPGTKCCKKP	-3.9	0.16	*Homo sapiens*
**Elafin**	1-50	2R3L	50	AQEPVKGPVSTKPGSCCCCRNPPNLMACCSGEGMACCPGIKKDTDKLRIL	-2.9	0.16	*Homo sapiens*
**Tricyclon A**	1-33	1YP8	33	CGESCFLGTCYTKGCSCGEWKLCYGTNGGTIFD	-5.3	0.15	*Viola tricolor*
**Kalata B8**	1-29	1K48	28	GSVLNCGETCLLGTCYTTGCTCNKYRVCTKD	-5.1	0.14	*Oldenlandia affinis*
**Palicourein**	1-37	1R1F	37	TFCGETCRVIPVCTYSAALGCTCDDRSDGLCKRNGDP	-4.2	0.14	*Palicourea condensata*
**RatNP-1**	1-30	Q62716	30	TCYCRRTRCGFRERLSGACGYRGRIYRLCC	-4.3	0.13	*Rattus norvegicus*
**Vhl-1**	1-31	1ZA8	31	CGESCAMISFCFTEVIGCSCKNKVCYLNSIS	-4.2	0.13	*Viola hederacea*
**Cycloviolacin O14**	1-31	2GJ0	31	GSIPACGESCFKGKCYTPGCSCSKYPLCAKN	-5.3	0.13	*Viola odorata*
**RatNP-3**	1-29	Q62713	29	CSCRTSSCRFGERLSGACRLNGRIYRLCC	-4.3	0.10	*Rattus norvegicus*
**Kalata B2**	1-29	1PT4	29	CGETCFGGTCNTPGCSCTWPICTRDGLPV	-5.2	0.10	*Oldenlandia affinis*
**Neutrophil defensin 4**	1-31	1ZMM	31	VCSCRLVFCRRTELRVGNCLIGGVSFTYCCT	-4.7	0.10	*Homo sapiens*
**Human neutrophil peptide-4**	1-31	1ZMM	31	VCSCRLVFCRRTELRVGNCLIGGVSFTYCCT	-4.7	0.097	*Homo sapiens*
**mBD-1**	1-36	P56386	36	DQYKCLQHGGFCLRSSCPSNTKLQGTCKPDKPNCCK	-3.1	0.08	*Mus musculus*
**Kalata B1**	1-29	4TTM	29	GLPVCGETCVGGTCNTPGCTCSWPVCTRN	-6.0	0.07	*Oldenlandia affinis*
**Circulin A**	1-30	1BH4	30	CGESCVWIPCISAALGCSCKNKVCYRNGIP	-3.9	0.07	*Chassalia parviflora*
**RNase 3 (alpha)**	6-35	1H1H	30	FTRAQWFAIQHISLNPPRCTIAMRAINNYR	-10.8	0.07	*Homo sapiens*
**Human neutrophil peptide-1**	1-27	2PM1	27	ACYCRIPACIAGEYGTCIYQGLWAFCC	-4.7	0.07	*Homo sapiens*
**HNP1**	1-27	2PM1	27	ACYCRIPACIAGEYGTCIYQGLWAFCC	-4.7	0.07	*Homo sapiens*
**Subtilosin A**	1-37	1PXQ	37	NKGCATCSIGAACLVDGPIPDFEIAGAGLGLWG	-5.1	0.05	*Bacillus subtilis*

**RNase 3 (alpha-beta)**	21-46	1H1H	26	PPRCTIAMRAINNYRWRCKNQNTFLR	-12.9	0.04	*Homo sapiens*

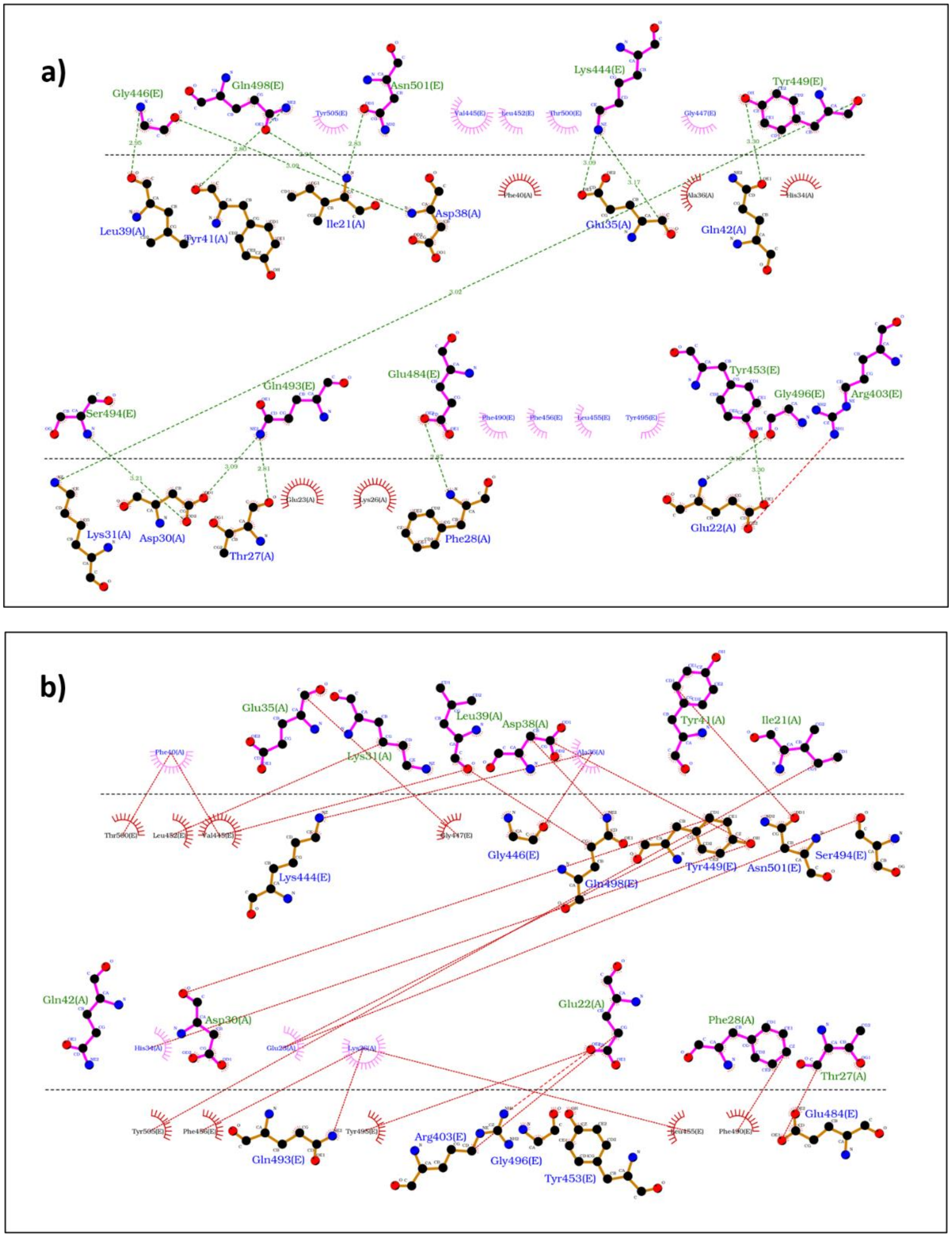

Figure S1: ACE2 docked to the SARS-CoV-2 RBD. (a) Interaction of amino acids by hydrogen bond (green dash line) and (b) representative hydrophobic interactions (red dash line) between ACE2 and RBD.

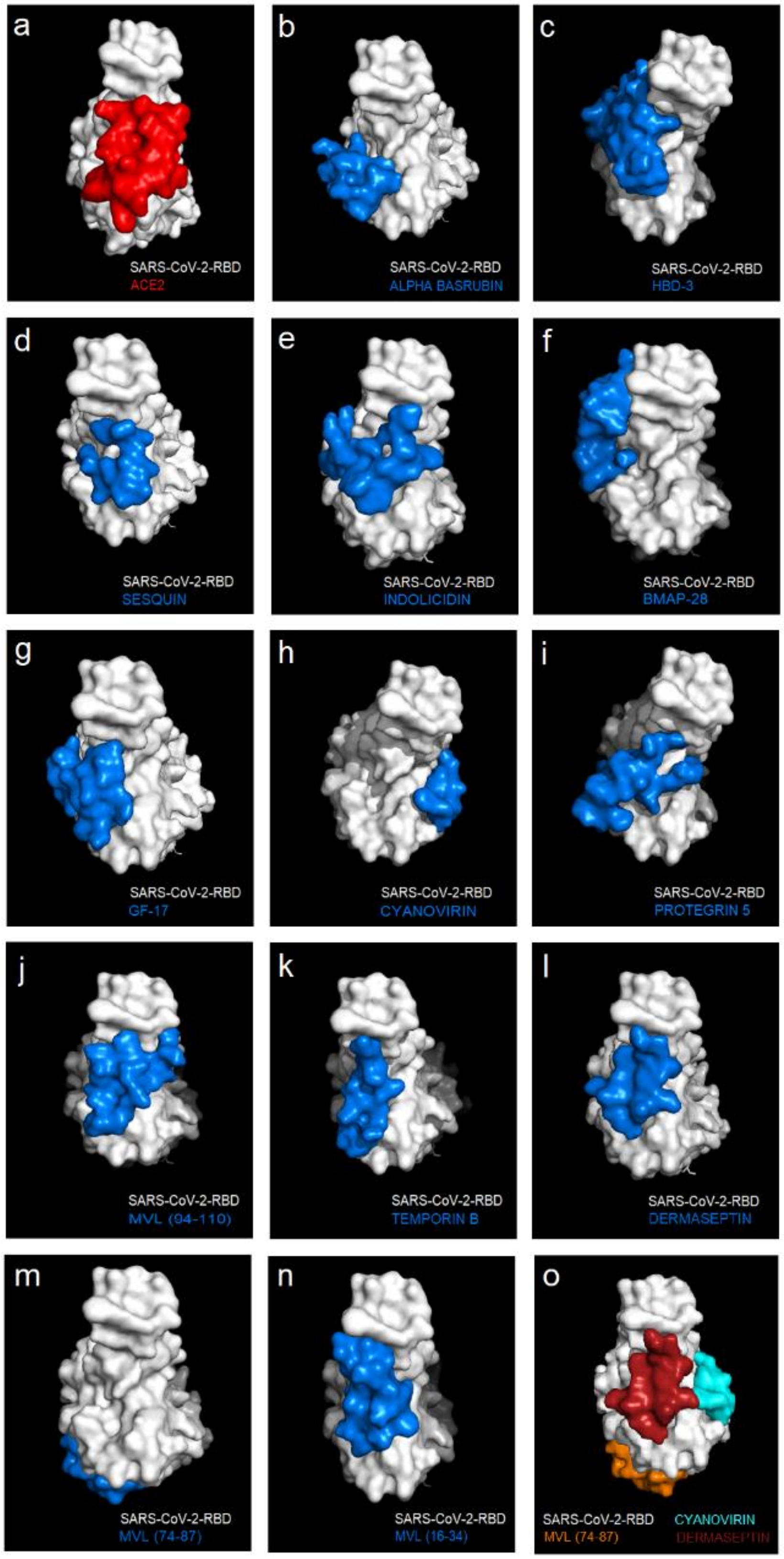

Figure S2: Peptide candidates (blue) docked to the SARS-CoV-2 RBD (white) with the highest binding affinities. (a) ACE2 (red). (b) Alpha basrubrin. (c) HBD-3. (d) Sesquin. (e) Indolicidin. (f) BMAP-28. (g) GF-17. (h) Cyanovirin. (i) Protegrin 5. (j) MVL 94-110. (k) Temporin-B. (l) Dermaseptin S4. (m) MVL (74–87). (n) MVL (16–34). (o) SARS-CoV-2 RBD (white) docked to three non-competing peptides.

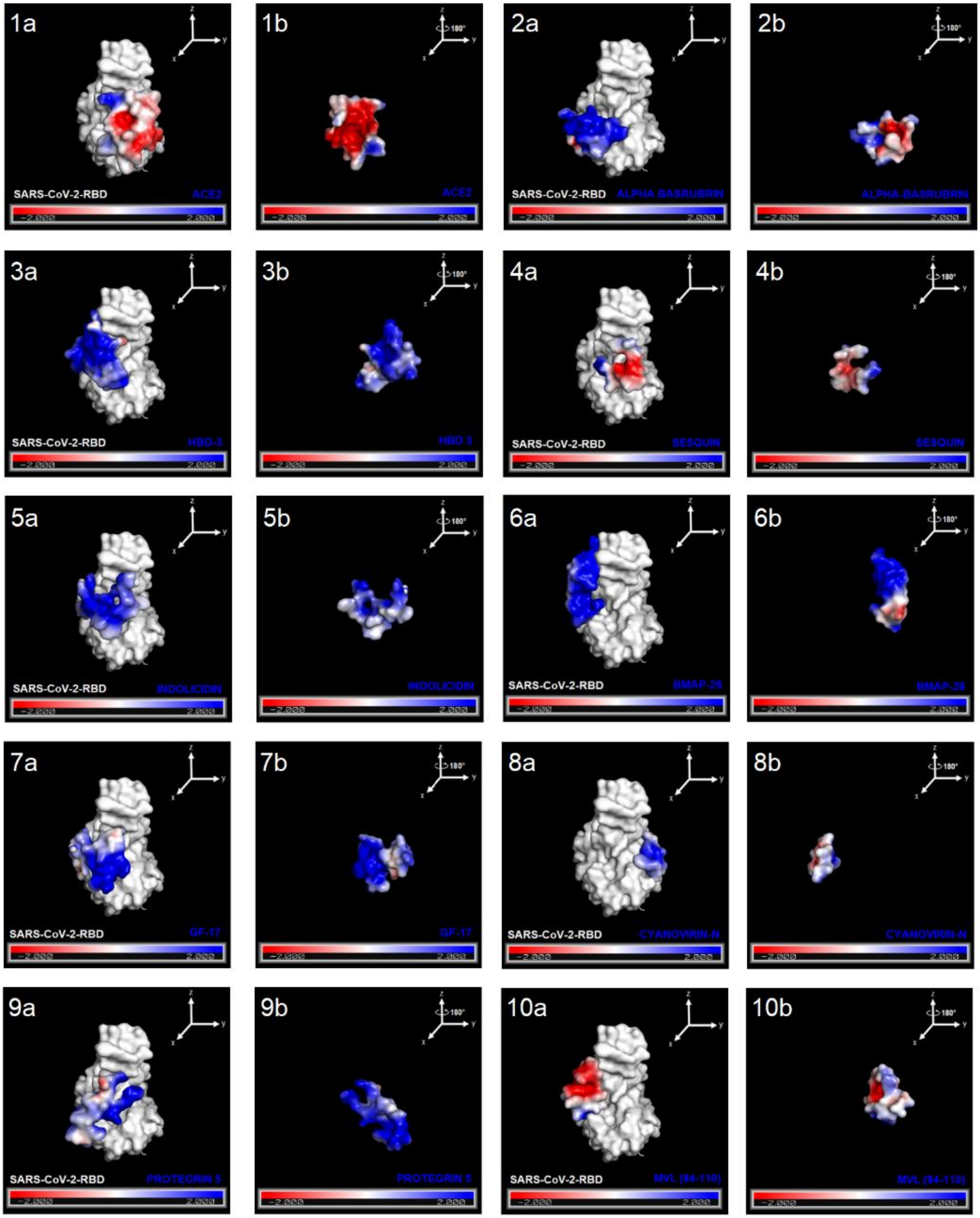
1a
SARS-CoV-2-RBD
ACE2
1b
ACE2
2a
SARS-CoV-2-RBD
ALPHA BASRUBRIN
2b
ALPHA-BASRUBRIN
3a
SARS-CoV-2-RBD
HBD-3
3b
HBD 3
4a
SARS-CoV-2-RBD
SESQUIN
4b
SESQUIN
5a
SARS-CoV-2-RBD
INDOLICIDIN
5b
INDOLICIDIN
6a
SARS-CoV-2-RBD
BMAP-28
6b
BMAP-28
7a
SARS-CoV-2-RBD
GF-17
7b
GF-17
8a
SARS-CoV-2-RBD
CYANOVIRIN-N
8b
CYANOVIRIN-N
9a
SARS-CoV-2-RBD
PROTEGRIN 5
9b
PROTEGRIN 5
10a
SARS-CoV-2-RBD
MVL (94-110)
10b
MVL (94-110)
-2.000
2.000
180°

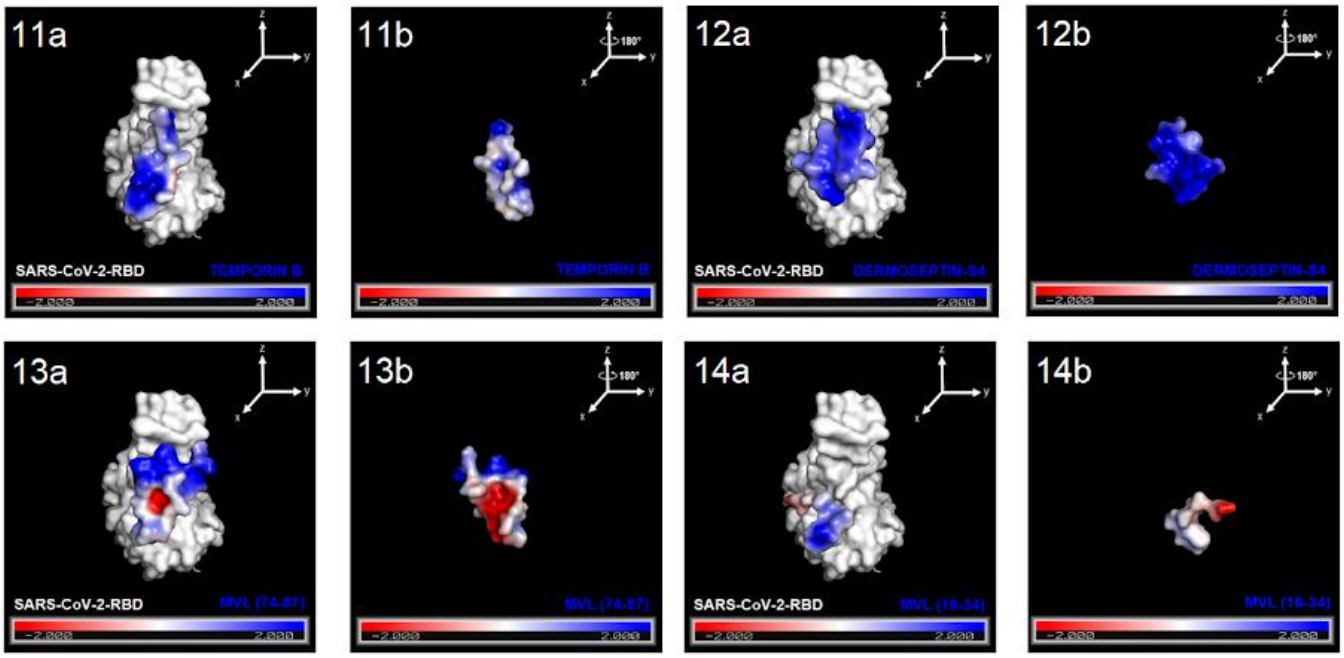

Figure S3: Distribution of electrostatic potential on the surface of APD peptide candidates docked in SARS-CoV-2 RBD. (1a,b) ACE2. (2a,b) Alpha basrubrin. (3a,b) HBD-3. (4a,b) Sesquin. (5a,b) Indolicidin. (6a,b) BMAP-28. (7a,b) GF-17. (8a,b) Cyanovirin-N (70–80). (9a,b) Protegrin 5. (10a,b) MVL (94–110). (11a,b) Temporin B. (12a,b) Dermaseptin-S4. (13a,b) MVL (74-87). (14a,b) MVL (16–34). The potential distribution was calculated by using the APBS module in PyMOL. The values range from −2 (red) over 0 (white) to +2 (blue). The orientation of the peptides in image (b) are rotated by about 180° along the *z*-axis of image (a) to show the peptide surface that binds to the RBD receptor.

Table S3: Hydrogen bonds and hydrophobic interactions of potential peptide candidates against SARS-CoV-2 RBD obtained by LigPlot+.

Peptide	Sequence fragment	PDB/ *UNIPRO*	Residues	Hydrogen bonds	Representative hydrophobic interactions	Isoelectric point (pH)
**Alpha Basrubrin**	1-20	*P83186*	20	19	30	6.34
**Human Beta Defensin 3**	27-44	1KJ6	19	18	30	10.41
**Sesquin**	1-10	*P84868*	10	8	17	3.93
**Indolicidin**	1-13	1G89	13	10	31	12.13
**BMAP-28**	1-18	2NDC	18	14	28	11.91
**GF-17**	1-17	2L5M	17	13	23	11.88
**Cyanovirin-N (70-80)**	70-80	2EZM	10	8	21	8.89
**Protegrin 5**	1-18	2NC7	18	13	21	9.67
**MVL (94-110)**	94-110	1ZHS	17	12	22	10.11
**Temporin B**	1-13	6GIL	13	9	19	10.12
**Dermaseptin-S4**	1-13	2DD6	13	9	18	11.17
**MVL (74-87)**	74-87	1ZHS	14	9	18	3.93
**MVL (16-34)**	16-34	1ZHS	19	12	31	7.86
**ACE2**	21-44	6VYB	24	14	24	4.0

Table S4: Immunogenicity analysis of peptides against Human MHC I to determine the number of alleles with IC50 < 50nM and IC50 < 500 nM concentrations.

Peptide	Sequence	No. Alleles IC50 < 50 nM	No. Alleles 50 < IC50 < 500 nM
**LI3**	WLNHSNNQTWDDWIDQDTQD	0	0
**CD3**	THDKTQHGQDQDQTTGQDWQQQ	0	0
**H3**	SGLTSWNDDNTQETWDQTTG	0	0
**P1**	LWRLGPDQTWGPTKRGPDWQ	0	0
**P1a**	THDLWDQDDDDTTGDYIQSQ	0	0
**P3a**	THDLWRQDRTGDRTDYIQSQ	0	0
**P9**	THRLWDQRRDTTDGSYIQSQ	0	0
**P13**	THRLWDQRDTTGRQSYDISQ	0	0
**P14**	THRLWRQRDTTGDSYIDQSQ	0	0
**P15**	THRLWRQRTDTGDSYIDQSQ	0	0
**Cyanovirin-N**	AAECKTRAQQ	0	0
**Sesquin**	KTCENLADTY	0	0
**Alpha-Basrubrin**	GADFQECMKEHSQKQHQHQG	0	0
**LD2**	GLTQIQTLNSLRRWDDTRRT	0	1
**CD2**	THDKTQHGQDQRQTTGQDWQQQ	0	1
**H1**	SGLTSWNRRNTQETWRQTTG	0	1
**H2**	SGLTSWNDRNTQETWRQTTG	0	1
**P2a**	THDLWRQDRDRTTGDYIQSQ	0	1
**P3**	LDYGGNQDLGGYTSLGGTDL	0	1
**P7**	THRLWDQRDRDTTGRYIQSQ	0	1
**P8**	THRLWDQRDRDTTGSYIQSQ	0	1
**P10**	THRLWDQRDTTRDGSYIQSQ	0	1
**P11**	THRLWDQRDTTGRDSYIQSQ	0	1
**P12**	THRLWDQRDTTGRQSYIDSQ	0	1
**LD3**	GLTQIQTLNSLDDWDDTDDT	1	1
**Temporin B**	LLPIVGNLLKSLL	0	1
**MVL (74-87)**	NDEAQKLGPQIAAS	0	1
**L3**	LSSQQTLDDWIDQDTGQDWY	0	2
**P4**	NDYGGNQLGSGYTSLGGTDN	0	2
**P4a**	THDLWRQDTGRDRTDYIQSQ	0	2
**P17**	THRLWRQRTTGSDYDIWDSQ	0	2
**CD1**	THRKTQHGQRQRQTTGQRWQQQ	1	2
**L2**	LSSQQTLDRWIRQDTGQDWY	1	2
**P16**	THRLWRQRTTGSDYDIQDSQ	1	2
**P8a**	THDLWDQDTGSRTRYWQRIS	2	2

**P9a**	THDLWDQDTGSRTYRWQRIS	2	2
**ACE2**	IEEQAKTFLDKFNHEAEDLFYQSS	3	2
**LI2**	WLNHSNNQTWDRWIDQRTQD	0	3
**P5**	NDYGGNQLDGGYTSLGGTDN	0	3
**P6a**	THDLWDQDTGRDTRYRYQSQ	0	3
**P6**	THRLWRQRRRRTTGRYIQSQ	1	3
**P7a**	THDLWDQDTGSRTRYRIQSQ	1	3
**P5a**	THDLWDQDTGRTRTYIQSRQ	2	3
**MVL (16-34)**	AEAQQVGPKIAAAHQGNFT	0	3
**GF-17**	GFKRIVQRIKDFLRNLV	2	3
**MVL (94-110)**	GQWRTIVEGVMSVIQIK	5	3
**Dermaseptin-S4**	ALWKTLLKKVLKA	5	3
**LI1**	WLNHSNNQTWRRWIRQRTQR	5	4
**L1**	LSSQQTLRRWIRQRTGQRWY	5	5
**Protegrin 5**	RGGRLCYCRPRFCVCVGR	0	5
**Indolicidin**	ILPWKWPWWPWRR	1	5
**Human Beta Defensin 3**	EEQIGKCSTRGRKCCRRKK	0	6
**P10a**	DTHDLTDQWGSWGYRTQRISR	2	8
**LD1**	GLTQIQTLNSLRRWIRTRRT	5	10
**BMAP-28**	GGLRSLGRKILRAWKKYGPIIVPIIRIG	3	12

Table S5: Contact area of peptides docked on SARS-CoV-2 RBDDE and RBDM variants.

RBDδ		RBDm	
**OAP**	**Ac**	**OAP**	**Ac**
**P1**	1039.78	**P1**	870.44
**ACE2**	1245.96	**ACE2**	1222.81
**Cyanovirin-N (70-80)**	604.08	**Cyanovirin-N (70-80)**	670.43
**Liso1_20**	919.27	**Liso1_20**	4915.35
**Liso111_130**	998.72	**Liso111_130**	4984.20
**Liso61_80**	732.16	**Liso61_80**	4974.99
**MVL (16-34)**	890.06	**MVL (16-34)**	782.62
**P10**	924.60	**P10**	856.24
**P11**	984.05	**P11**	959.13
**P12**	1171.33	**P12**	791.97
**P13**	854.73	**P13**	938.85
**P15**	937.56	**P15**	1039.63
**P17**	1165.96	**P17**	856.47
**P2a**	770.42	**P2a**	957.17
**P4a**	1064.26	**P4a**	1093.94
**P6a**	944.48	**P6a**	1043.39
**P7**	1037.59	**P7**	941.23
**P8**	1118.28	**P8**	897.82
**P9**	841.20	**P9**	972.60
**PH1**	871.82	**PH1**	997.86
**PH2**	833.10	**PH2**	921.22
**Sesquin**	641.88	**Sesquin**	684.09
**TemporinB**	681.97	**TemporinB**	704.49
